# Distinct roles of KLF4 in mesenchymal cell subtypes during lung fibrogenesis

**DOI:** 10.1038/s41467-021-27499-8

**Published:** 2021-12-10

**Authors:** Rachana R. Chandran, Yi Xie, Eunate Gallardo-Vara, Taylor Adams, Rolando Garcia-Milian, Inamul Kabir, Abdul Q. Sheikh, Naftali Kaminski, Kathleen A. Martin, Erica L. Herzog, Daniel M. Greif

**Affiliations:** 1 Yale Cardiovascular Research Center, Section of Cardiovascular Medicine, Department of Internal Medicine, Yale University School of Medicine, New Haven, CT 06511 USA; 2 Department of Genetics, Yale University School of Medicine, New Haven, CT 06520 USA; 3 Department of Pharmacology, Yale University School of Medicine, New Haven, CT 06520 USA; 4 Section of Pulmonary, Critical Care and Sleep Medicine, Department of Internal Medicine, Yale University School of Medicine, New Haven, CT 06520 USA; 5 Bioinformatics Support Program, Yale University School of Medicine, New Haven, CT 06520 USA; 6 Department of Pathology, Yale University School of Medicine, New Haven, CT 06520 USA; 7 Present Address: Pfizer, 610 Main Street, Cambridge, MA 02139 USA

**Keywords:** Respiratory tract diseases, Pathogenesis, Mesenchymal stem cells

## Abstract

During lung fibrosis, the epithelium induces signaling to underlying mesenchyme to generate excess myofibroblasts and extracellular matrix; herein, we focus on signaling in the mesenchyme. Our studies indicate that platelet-derived growth factor receptor (PDGFR)-β^+^ cells are the predominant source of myofibroblasts and Kruppel-like factor (KLF) 4 is upregulated in PDGFR-β^+^ cells, inducing TGFβ pathway signaling and fibrosis. In fibrotic lung patches, KLF4 is down-regulated, suggesting KLF4 levels decrease as PDGFR-β^+^ cells transition into myofibroblasts. In contrast to PDGFR-β^+^ cells, KLF4 reduction in α-smooth muscle actin (SMA)^+^ cells non-cell autonomously exacerbates lung fibrosis by inducing macrophage accumulation and pro-fibrotic effects of PDGFR-β^+^ cells via a Forkhead box M1 to C-C chemokine ligand 2—receptor 2 pathway. Taken together, in the context of lung fibrosis, our results indicate that KLF4 plays opposing roles in PDGFR-β^+^ cells and SMA^+^ cells and highlight the importance of further studies of interactions between distinct mesenchymal cell types.

## Abstract (web summary)

The pluripotency factor KLF4 has been described as pro-fibrotic or anti-fibrotic in various diseases. Herein, the authors show that during lung fibrosis, these distinct effects can be attributed to mesenchymal cell-type specific functions of KLF4.

## Introduction

Pulmonary fibrosis is a devastating disease that results from insults to the lung and is characterized by the exaggerated production and impaired turnover of extracellular matrix (ECM) leading to architectural distortion and impaired gas transfer in the lung^1^. Pulmonary fibrosis has diverse known causes including exposure to toxins or bleomycin or is idiopathic (IPF). IPF, the most common form of interstitial lung disease, is particularly lethal causing 40,000 deaths annually in the USA alone, and the median survival after initial diagnosis is a dismal ~3 years^2,3^. The course of IPF is highly variable as for unclear reasons, some patients experience rapid deterioration in lung function while others experience more gradual decline^4,5^. The success of two drugs, pirfenidone and nintedanib, in delaying disease progression indicates that the clinical course can be altered, but the benefit of these agents is unpredictable, at best modest, and accompanied by significant toxicities^2,6^.

These major limitations in prognostication and treatment options for pulmonary fibrosis largely stem from our inadequate understanding of the underlying cellular and molecular mechanisms driving pathogenesis. In the normal lung, α-smooth muscle actin (SMA) is expressed by airway and vascular smooth muscle cells (SMCs) and the very rare alveolar myofibroblasts^7^. A hallmark of pulmonary fibrosis is a marked increase in myofibroblasts which are implicated in pathological contraction of lung tissue and may contribute to synthesis of excessive ECM^2,3,8^. The current paradigm of IPF pathogenesis suggests that repeated alveolar epithelial cell injury activates the underlying mesenchyme and results in accumulation of excess myofibroblasts and ECM through as yet undefined mechanisms^9,10^. This paradigm points towards signaling from the epithelium to the mesenchyme; however, in the context of lung fibrosis, little attention has been paid to the crosstalk between mesenchymal cell types (e.g., signaling between SMCs and fibroblasts). In addition, the role of SMCs in general during fibrosis pathogenesis is not well understood.

The cellular origins of myofibroblasts in lung fibrosis are incompletely defined although multiple postembryonic mesenchymal cell populations are believed to be dominant sources. Relevant studies have relied heavily on the bleomycin mouse model of lung fibrosis which shares key histopathological findings with human IPF, including excessive and aberrant myofibroblast accumulation and collagen deposition and obliteration of alveoli^11,12^. In the context of bleomycin exposure, fate mapping studies have implicated ADRP^+^ lipofibroblasts^13^, Axin2^+^ progenitors^14^, Gli1^+^ mesenchymal stem cell-like cells^15^ and FoxD1^+^ progenitor-derived pericytes^16^ as sources of substantial myofibroblasts whereas another study indicates that “pericyte-like cells” expressing NG2 or FoxJ1 do not contribute^17^. The mesenchyme marker platelet-derived growth factor receptor (PDGFR)-β is expressed by many of these mesenchymal cell types, including fibroblasts, pericytes, Gli1^+^ mesenchymal stem cell (MSC)-like cells and Axin2^+^ progenitors^14,15,18^, but no prior studies have utilized cell labeling driven by an inducible *Pdgfrb* promoter for fate mapping in lung fibrosis.

Beyond cellular origins, insights into molecular pathways critical for mesenchymal cell transitions during the pathogenesis of lung fibrosis are needed. Treatment with an anti-PDGFR-β blocking antibody has been shown to attenuate bleomycin-induced lung fibrosis and proliferation of cells in the inter-alveolar space^19^. Herein, we focus on Kruppel-like factor 4 (KLF4), a pluripotency transcription factor (TF) integral in cell fate and reprogramming^20–22^. KLF4 is expressed in diverse cell types found in the lung, including epithelial cells, leukocytes and fibroblasts as well as in the context of disease, in perivascular cells (i.e., SMCs and/or pericytes)^23–26^. Myofibroblasts express SMC markers (e.g., SMA), and in SMCs, studies indicate that KLF4 induces dedifferentiation of SMCs^27,28^; in contrast, however, there is no consensus regarding the role of KLF4 in myofibroblast differentiation and fibrosis. For instance, in vimentin^+^ cells isolated from neonatal mouse hearts and cultured in conditions to eliminate cardiomyocytes, KLF4 induces SMA levels and potentiates angiotensin II-induced collagen levels^29^; however, KLF4 function in renal fibrosis is controversial with studies suggesting that KLF4 is either protective or deleterious^30^. In the lung, KLF4 has been implicated in the normal perinatal accumulation of myofibroblasts as *Klf4*^*(−/−)*^ neonates lack alveolar myofibroblasts, and expression of KLF4 in lung fibroblasts isolated from *Klf4* null mice induces expression of SMA, collagen and fibronectin^23^. In contrast, a study of cultured cells with fibroblast-like morphology isolated from the rodent lung reported that KLF4 binds SMAD3 and thereby, inhibits activation of the *Acta2* promoter^31,32^. Finally, a recent study reported that KLF4 overexpression by unspecified cells in the mouse attenuates bleomycin-induced lung fibrosis^33^.

Herein, we use conditional genetic labeling to demonstrate that select PDGFR-β^+^ cells clonally expand and give rise to the majority of myofibroblasts in bleomycin-induced lung fibrosis whereas only rare myofibroblasts derive from preexisting SMA^+^ cells. Furthermore, these studies delineate mesenchymal cell-type-specific roles of KLF4 in lung fibrosis. KLF4 expression is upregulated in PDGFR-β^+^ cells and their lineage during disease pathogenesis. Reduction of KLF4 in PDGFR-β^+^ cells attenuates fibrosis, at least in part by downregulating transforming growth factor (TGF)β pathway activation and thereby, reducing ECM levels. In stark contrast, *Klf4* deletion in SMA^+^ cells exacerbates fibrosis, and the data indicate that this effect is largely noncell autonomous. Indeed, decrease of KLF4 levels in SMA^+^ cells enhances C–C motif chemokine ligand 2 (CCL2) levels via Forkhead box M1 (FOXM1) upregulation, induces macrophage recruitment and stimulates profibrotic transformation of PDGFR-β^+^ cells via the cognate receptor C–C motif chemokine receptor 2 (CCR2). Thus, KLF4 plays critical and contrasting roles in the regulation of lung fibrosis depending on the specific mesenchymal cell type: profibrotic in PDGFR-β^+^ cells and antifibrotic in SMA^+^ cells.

## Results

### PDGFR-β^+^ cells are the primary source of lung myofibroblasts

The normal adult lung has only very rare SMA^+^ myofibroblasts^7,34,35^, but this cell type accumulates in the fibrotic lung and is considered to play key pathological roles. PDGFR-β is expressed in diverse cell types, including fibroblasts, pericytes, Axin2^+^ myofibrogenic progenitors, Gli1^+^ MSC-like cells and select vascular SMCs, of which all except the latter have been implicated as a source of lung myofibroblasts^7,14,15,18,24^. Almost all SMA^+^ lung myofibroblasts in bleomycin-induced fibrosis express PDGFR-β^19^ (Supplementary Fig. 1), and thus, studies with the constitutive *Pdgfrb-Cre* in this context are not informative with regard to cellular origins of myofibroblasts^36^. No prior studies have conditionally labeled PDGFR-β^+^ cells (with for instance, *Pdgfrb-CreER*^*T2*^) to follow their fate during the course of lung fibrosis. To initially assess what cell types in the lung are marked by *Pdgfrb-CreER*^*T2*^ under basal conditions, *Pdgfrb-CreER*^*T2*^*, ROSA26R*^*(Zs/+)*^ mice were induced with tamoxifen (1 mg/day for 5 days) and then rested for 5 days (Supplementary Fig. 2). The lungs were subjected to 10X Genomics single-cell (sc) RNA library construction followed by sequencing (seq), and scRNA-seq data were aligned to a modified version of the mouse transcriptome mm10, including the ZsGreen1 (Zs) sequence. Cells were annotated and clustered in concordance with a recent scRNA-seq analysis of collagen-producing lung cells^37^, and a uniform manifold approximation and projection was used to visualize Zs and Pdgfrb expression in lung stromal cells (Supplementary Figs. 3a–c and 4). These results suggest that both Pdgfrb and Zs are expressed in diverse populations of fibroblasts as well as pericytes in the *Pdgfrb-CreER*^*T2*^*, ROSA26R*^*(Zs/+)*^ lung. A previous scRNA-seq analysis demonstrates Pdgfrb expression in multiple similar lung fibroblast populations^38^.

In addition, the expression patterns of Gli1, Pdgfra and Axin2 were evaluated (Supplementary Fig. 3d–f). Prior immunohistochemical analysis indicates that Gli1^+^ cells express PDGFR-α and PDGFR-β^15^, and our scRNA-seq shows that both Gli1 and Pdgfra are primarily expressed in adventitial and alveolar fibroblasts, clusters that are largely Pdgfrb^+^. In contrast, consistent with prior studies^14,37^, Axin2 is broadly expressed. Peribronchiolar Axin2^+^ cells that are predominantly PDGFR-α^-^PDGFR-β^+^ are implicated as myofibrogenic progenitor cells, and these Axin2^+^ cells have relatively high mRNA levels of Acta2, transgelin and desmin^14^. Based on this expression pattern and our scRNA-seq data (Supplementary Fig. 3b, e–i), Axin2^+^ myofibrogenic progenitors may be enriched in our peribronchial fibroblast/SMC cluster.

**Fig. 1:**
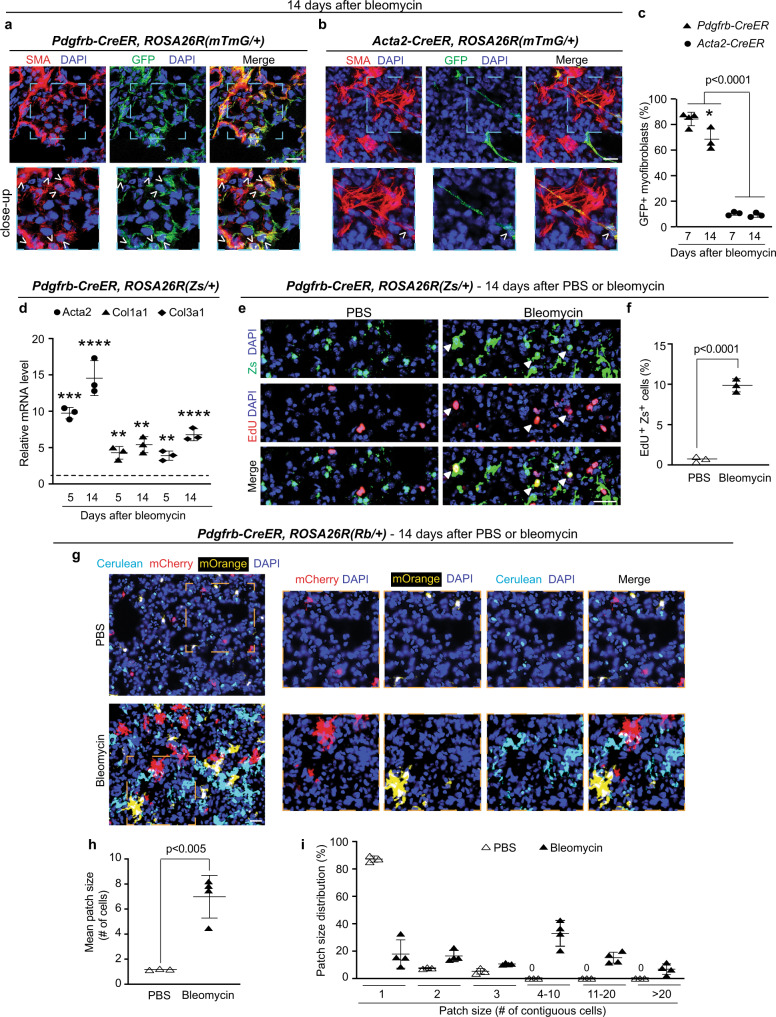
PDGFR-β^+^ cells give rise to myofibroblasts, express collagen and clonally expand during lung fibrogenesis. **a–c** *ROSA26R*^(*mTmG*/+)^ mice carrying *Pdgfrb-CreER*^*T2*^ or *Acta2*-*CreER*^*T2*^ were induced with tamoxifen, rested and subjected to an orotracheal bleomycin dose. Seven or fourteen days later, mice were euthanized, and lung vibratome sections were stained for SMA and GFP (fate marker). Images (**a**, **b**) with close-ups of boxed regions below. Arrowheads indicate GFP^+^ myofibroblasts. Percent of myofibroblasts that are GFP^+^ (**c**). For each genotype and time point, *n* = 3 mice except *n* = 4 mice for *Pdgfrb-CreER*^*T2*^ at 7 days, 6–12 sections per mouse, average of 58 myofibroblasts per section; * vs. *Pdgfrb-CreER*^*T2*^ at 7 days, *p* = 0.0143. **d** *Pdgfrb-CreER*^*T2*^*, ROSA26R*^*(Zs/+)*^ mice were induced with tamoxifen, rested, treated or not treated with bleomycin, and 5 or 14 days later, Zs^+^ cells were isolated by FACS and subjected to qRT-PCR. Levels of indicated transcripts are relative to Gapdh and normalized to no treatment. For each treatment group and time point, *n* = 3 mice in triplicate; **, Col1a1, day 5 (*p* = 0.0071), Col1a1, day 14 (*p* = 0.0016), Col3a1, day 5 (*p* = 0.0027), ***p = 0.0008, ****p < 0.0001 vs. no treatment, respectively. **e, f** *Pdgfrb-CreER*^*T2*^*, ROSA26R*^*(Zs/+)*^ were labeled with tamoxifen, rested and treated with PBS or bleomycin. On the evening of the 13th day thereafter, EdU was injected, and 12 h later, mice were euthanized. Lung cryosections were stained for EdU and directly imaged for Zs (**e**) with closed arrowheads indicating EdU^+^Zs^+^ cells. Percent of Zs^+^ cells that are EdU^+^ is quantified (**f**). For each treatment group, *n* = 3 mice, three sections per mouse, average 90 Zs^+^ cells per section. **g–i** *Pdgfrb-CreER*^*T2*^*, ROSA26R*^*(Rb/+)*^ were labeled with tamoxifen, rested, subjected to an orotracheal dose of PBS or bleomycin and euthanized 14 days later. Lung cryosections were directly imaged for Rb colors (mCherry, mOrange, Cerulean) with boxed regions shown as close-ups [right; **g**]. In each patch, the number of contiguous labeled cells of the same color was scored and averaged (**h**; *p* = 0.0022) or binned into groups (**i**). *n* = 3 mice for PBS and *n* = 4 for bleomycin groups, three sections per mouse, average of 16 patches per section. One-way ANOVA with Tukey’s multiple comparisons test (**c, d**) and two-tailed Student’s *t* test (**f, h**). Data are averages ± SD. Source data are provided as a Source Data file. Scale bars, 25 μm.

We then proceeded to fate mapping of PDGFR-β^+^ cells during lung fibrosis. To this end, *Pdgfrb-CreER*^*T2*^*, ROSA26R*^*(mTmG/+)*^ mice were injected with tamoxifen and rested to mark PDGFR-β^+^ cells (Supplementary Fig. 5a, b) and then treated with a single orotracheal dose of phosphate-buffered saline (PBS) or bleomycin to induce lung fibrosis (Fig. 1a and Supplementary Fig. 5c, d). In order to ensure lung myofibroblast accumulation, mice were analyzed at 7 and 14 days following bleomycin administration (the latter is a time point of established fibrosis). Of the total lung myofibroblasts, PDGFR-β^+^ cells give rise to 84 ± 5% on day 7 following bleomycin administration and 68 ± 9% on day 14 (Fig. 1a, c). In contrast, fate mapping with *Acta2-CreER*^*T2*^, which in the normal lung marks airway and vascular SMCs as well as the very rare alveolar myofibroblasts, indicates that only 10 ± 2% of bleomycin-induced myofibroblasts derive from preexisting SMA^+^ cells (Fig. 1b, c and Supplementary Fig. 6). Interestingly, in mice exposed to hypoxia as an alternate model of lung pathology that induces myofibroblast accumulation—similar to results with bleomycin—PDGFR-β^+^ cells give rise to a substantial percentage of myofibroblasts (43 ± 6%) (Supplementary Fig. 7a) whereas SMA^+^ cells only contribute ~10%^7^. To complement and extend the immunohistochemical studies in lung fibrosis, following bleomycin treatment of tamoxifen-induced *Pdgfrb-CreER*^*T2*^*, ROSA26R*^*(Zs/+)*^ mice, lung Zs^+^ cells were isolated by fluorescence-activated cell sorting (FACS) and subjected to qRT-PCR. During lung fibrosis, the expression of Acta2 and collagen 1a1 (Col1a1) and 3a1 (Col3a1) in the PDGFR-β^+^ cell lineage is markedly increased (Fig. 1d).

### PDGFR-β^+^ cells proliferate and clonally expand during injury

Given that PDGFR-β^+^ cells are the main source of myofibroblasts in lung fibrosis, we next determined whether bleomycin induces PDGFR-β^+^ cell proliferation and if so, clonal expansion. *Pdgfrb-CreER*^*T2*^*, ROSA26R*^*(Zs/+)*^ mice were induced with tamoxifen, rested and then treated with a single dose of orotracheal PBS (control) or bleomycin. Fourteen days later, 5-ethynyl-2′-deoxyuridine (EdU) was administrated 12 h prior to euthanasia. Lung sections were stained for EdU and nuclei (DAPI) and directly imaged for Zs. Following bleomycin administration, 9.9 ± 0.8% of the PDGFR-β^+^ cell lineage is EdU^+^ in comparison to 0.7 ± 0.3% following PBS (Fig. 1e, f).

To examine the clonality and mixing of the PDGFR-β^+^ cell lineage in the fibrotic lung, *Pdgfrb-CreER*^*T2*^ mice carrying an allele of the multicolor Rainbow (Rb) Cre reporter, which upon recombination expresses Cerulean, mCherry or mOrange in a mutually exclusive manner^24,39^, were used. These mice were induced with tamoxifen, rested and then a single dose of orotracheal PBS or bleomycin was administered. At 14 days, the size of clonal patches in the lung parenchyma was scored based on contiguous cells labeled with the same fluorophore and then averaged or binned into patch size groups (Fig. 1g–i; see “Methods”). For mice treated with PBS or bleomycin, the mean patch size is 1.2 ± 0.1 or 7.0 ± 1.7 cells and the maximum patch size is 3 or 30 cells, respectively. Thus, upon bleomycin exposure, PDGFR-β^+^ cells undergo clonal expansion to form sizable congruent patches of cells that display limited mixing with other cells (Supplementary Fig. 8).

### KLF4 is upregulated in PDGFR-β^+^ cell lineage during lung injury

**Fig. 2:**
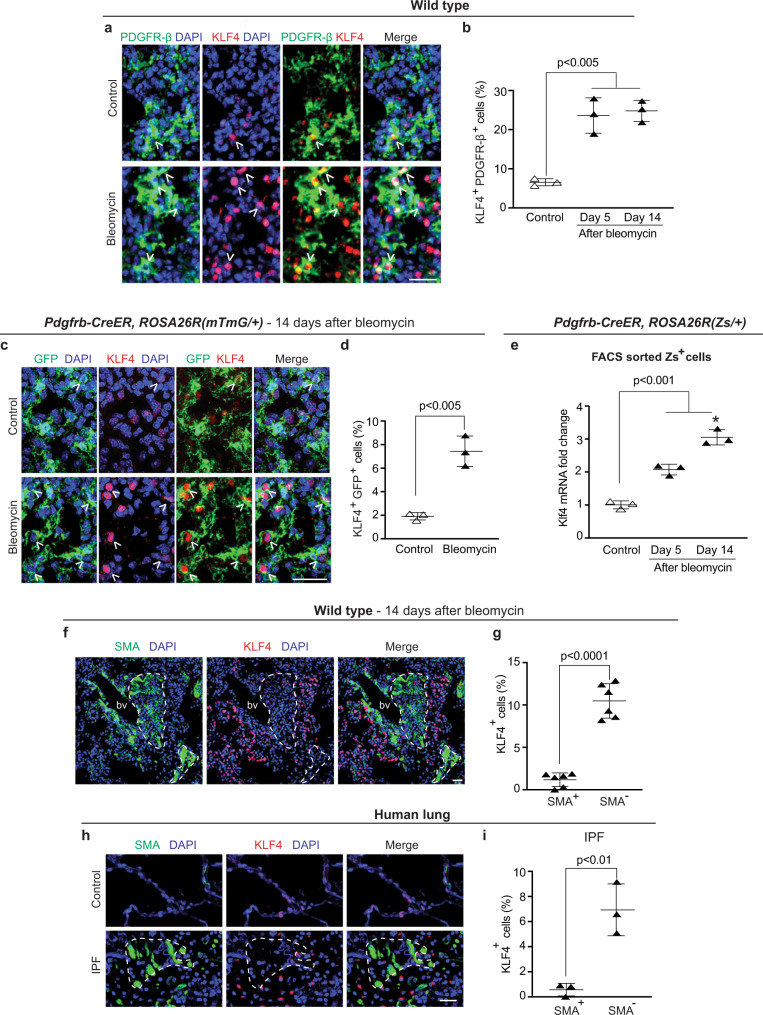
Klf4 is upregulated in PDGFR-β^+^ cells after bleomycin treatment. **a, b**, Wild-type mice were or were not subjected to an orotracheal dose of bleomycin, and 5 or 14 days thereafter, lung cryosections were stained for PDGFR-β and KLF4. Images at 14 days (**a**) were used to quantify the percent of PDGFR-β^+^ lung cells expressing KLF4 (**b**). For each treatment group, *n* = 3 mice, three sections per mouse and an average of 53 PDGFR-β^+^ cells per section. Day 5 (*p* = 0.0013), day 14 (*p* = 0.0009) vs. control. **c, d** *Pdgfrb-CreER*^*T2*^*, ROSA26R*^*(mTmG/+)*^ mice were induced with tamoxifen, rested and given an orotracheal dose of PBS or bleomycin. Fourteen days later, lung cryosections were stained for GFP (fate marker) and KLF4 (**c**), and the percent of GFP^+^ cells that are KLF4^+^ was quantified (**d**). For each treatment group, *n* = 3 mice, three sections per mouse and an average of 40 GFP^+^ cells per section. *p* = 0.002. **e** *Pdgfrb-CreER*^*T2*^*, ROSA26R*^*(Zs/+)*^ mice were induced with tamoxifen, rested, treated or not treated with orotracheal bleomycin, and 5 or 14 days later, Zs^+^ cells were isolated by FACS and subjected to qRT-PCR for Klf4 and Gapdh. Klf4 mRNA relative to Gapdh and normalized to untreated control is shown. For each treatment group, *n* = 3 mice with qRT-PCR done in triplicate. Day 5 (*p* = 0.0007), day 14 (*p* < 0.0001) vs. control; * vs. day 5, *p* = 0.012. **f–i** Lung stains for SMA and KLF4 of cryosections at 14 days after bleomycin-induction of wild-type mice (**f**) and of paraffin sections from human control and IPF patients (**h**). bv, blood vessel. Fibrotic areas demarked by dashed lines have minimal KLF4 staining. In **g** and **i**, the percent of SMA^+^ and SMA^-^ parenchymal cells that are KLF4^+^ is quantified from sections as in **f** and **h**; *n* = 6 mice, three sections per mouse, average of 178 SMA^+^ cells and 910 SMA^-^ cells per section, *p* < 0.0001 and *n* = 3 humans, three sections per human, average of 110 SMA^+^ cells and 729 SMA^-^ cells per section, *p* = 0.0066. One-way ANOVA with Tukey’s multiple comparisons test (**b, e**) and two-tailed Student’s *t* test (**d, g, i**). Data are averages ± SD. Source data are provided as a Source Data file. Scale bars, 25 μm.

We and others have studied the pluripotency factor KLF4 in the context of diverse relevant diseases, including pulmonary hypertension^7,24^, lung cancer^40^ and organ fibrosis^29,30,33^; however, the role of KLF4 in PDGFR-β^+^ cells and cells derived from PDGFR-β^+^ cells in fibrosis has not been investigated. Our scRNA-seq indicates that Klf4 is broadly expressed in the normal lung under basal conditions, including in Pdgfrb^+^ cells, and within the population of Pdgfrb^+^ cells, Klf4 is most highly expressed in the adventitial fibroblast cluster (Supplementary Fig. 3j). We next evaluated KLF4 expression in PDGFR-β^+^ cells during the course of fibrosis at day 5 (in the acute inflammation phase^11^) and at day 14. In bleomycin-treated wild-type mice, lung sections demonstrate a ~4-fold increase in the percent of PDGFR-β^+^ cells expressing KLF4 compared to untreated controls (Fig. 2a, b). Next, *Pdgfrb-CreER*^*T2*^*, ROSA26R*^*(mTmG/+)*^ mice were induced with tamoxifen, rested and then treated with a single orotracheal dose of PBS (control) or bleomycin. Fourteen or twenty-one days later, mice were euthanized and lungs were harvested, sectioned and stained for GFP, KLF4 and nuclei (DAPI). Compared to control, lung sections from bleomycin-treated mice demonstrate a ~3.5-fold increase in the percent of GFP^+^ cells expressing KLF4 at day 14 but no change at day 21 (Fig. 2c, d and Supplementary Fig. 9a, b). In addition to the substantial increase in the percent of PDGFR-β^+^ cells and PDGFR-β^+^ lineage cells expressing KLF4, the intensity of staining for KLF4 in these cells is markedly enhanced at day 14 after bleomycin (Fig. 2a, c). To further evaluate the expression level of Klf4 in the PDGFR-β^+^ cell lineage, tamoxifen-induced *Pdgfrb-CreER*^*T2*^*, ROSA26R*^*(Zs/+)*^ mice were or were not treated with bleomycin. At 5, 14 or 21 days thereafter, lung Zs^+^ cells were isolated by FACS and subjected to qRT-PCR for Klf4. In comparison to untreated mice, Klf4 transcript levels are increased by ~2-fold at day 5 and ~3-fold at day 14 and unchanged at day 21 (Fig. 2e and Supplementary Fig. 9c). In addition, we observed a marked reduction in KLF4 expression in SMA^+^ fibrotic patches in the lungs of bleomycin-treated mice and of humans with IPF (Fig. 2f–i and Supplementary Fig. 10). Thus, KLF4 expression is increased in PDGFR-β^+^ cells and PDGFR-β^+^ lineage cells during bleomycin-induced fibrosis, and we speculate these cells downregulate KLF4 as they differentiate into SMA^+^ cells in fibrotic patches of the lung.

### *Klf4* deletion in PDGFR-β^+^ cells mitigates pulmonary fibrosis

**Fig. 3:**
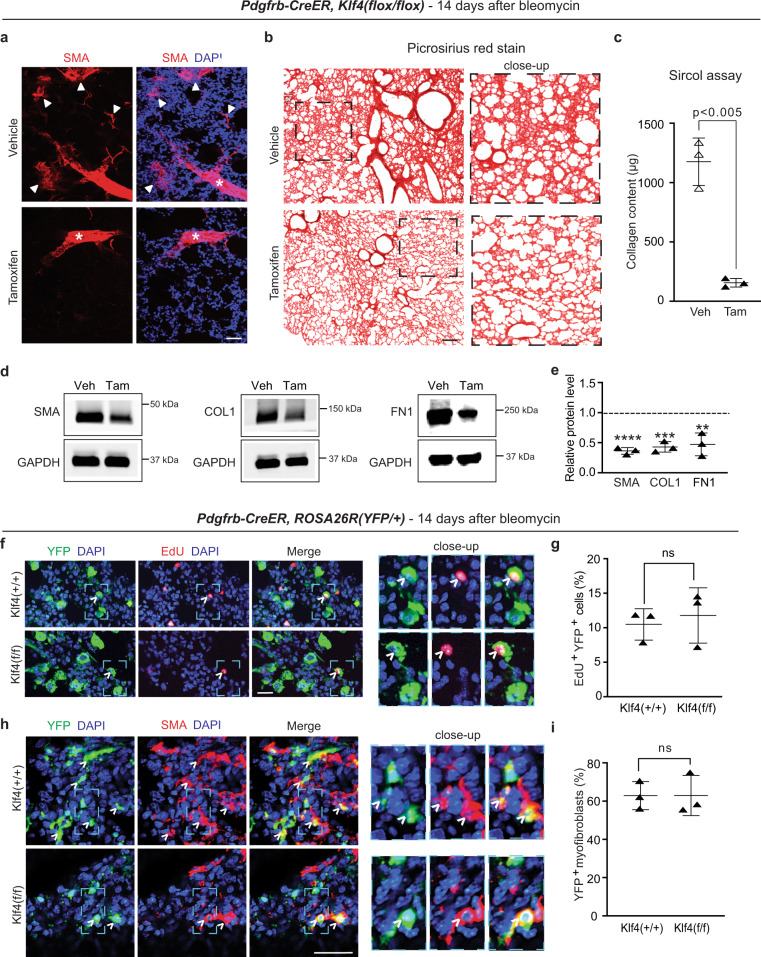
*Klf4* deletion in PDGFR-β^+^ cells attenuates bleomycin-induced myofibroblast accumulation and lung fibrosis. **a–e** *Pdgfrb*-*CreER*^*T2*^, *Klf4*^*(flox/flox)*^ mice were induced with either vehicle (Veh; corn oil) or tamoxifen (Tam), rested and treated with bleomycin. Lungs were analyzed 14 days later. Cryosections were stained for SMA and nuclei (DAPI) in (**a**) and with Picrosirius red (**b**) with dashed boxes shown as close-ups on the right. Closed arrowheads and asterisks indicate SMA^+^ fibrotic patches and blood vessels, respectively. *n* = 3 mice for vehicle and *n* = 6 mice for tamoxifen inductions. Collagen content of the right lung was measured using the Sircol assay (**c**); *n* = 3 mice in duplicate, *p* = 0.001. Western blot analysis of lung lysates for SMA, collagen 1 (COL1), fibronectin (FN1) is shown in **d** with densitometric analysis relative to GAPDH and normalized to vehicle (**e**). *n* = 3; **, ***, **** vs. vehicle, *p* = 0.0086, *p* = 0.0003, *p* < 0.0001, respectively. **f**–**i** *Pdgfrb-CreER*^*T2*^*, ROSA26R*^*(YFP/+)*^ mice carrying *Klf4*^*(flox/flox)*^ or wild type for *Klf4* were induced with tamoxifen, rested, treated with bleomycin and lungs were analyzed 14 days later. Mice were injected with EdU 12 h prior to euthanasia. Lung cryosections were stained for YFP (fate marker) and nuclei (DAPI) as well as for EdU (**f**) or SMA (**h**) with boxed regions shown as close-ups on the right. Open arrowheads indicate YFP^+^ cells that are also EdU^+^ (**f**) or SMA^+^ (**h**). The percentage of YFP^+^ cells that are EdU^+^ or are SMA^+^ myofibroblasts was quantified in **g** and **i**, respectively. ns not significant. For each genotype, we analyzed *n* = 3 mice, three sections per mouse, and on average 41 YFP^+^ cells (**g**) or 68 myofibroblasts (**i**) per section. Two-tailed Student’s *t* test was used for all analysis. Data are averages ± SD. Source data are provided as a Source Data file. Scale bars, 200 μm (**b**), 50 μm (**a**), 25 μm (**f**, **h**).

Given the early upregulation of KLF4 in PDGFR-β^+^ cells following bleomycin treatment, we hypothesized that KLF4 plays a role in the profibrotic transformation of PDGFR-β^+^ cells. To test this hypothesis, *Pdgfrb-CreER*^*T2*^*, Klf4*^*(flox/flox)*^ mice were injected with tamoxifen to delete *Klf4* in PDGFR-β^+^ cells (Supplementary Fig. 11a) or corn oil (vehicle), rested and subjected to a single orotracheal dose of bleomycin. Fourteen days later, mice were euthanized, and lungs were harvested and analyzed. Deletion of *Klf4* in PDGFR-β^+^ cells considerably reduces bleomycin-induced lung accumulation of excess SMA^+^ myofibroblasts (Fig. 3a) and collagen as demonstrated by qualitative Picrosirius red staining (Fig. 3b) and the quantitative Sircol assay (Fig. 3c). Western blot analysis of whole lung lysates also revealed a reduction in collagen 1 (COL1), fibronectin 1 (FN1) and SMA protein levels (Fig. 3d, e). These data indicate that KLF4 expression in PDGFR-β^+^ cells is a key factor inducing myofibroblast accumulation and fibrosis in the lung upon bleomycin exposure. Once again, similar results were obtained with hypoxia as deletion of *Klf4* in PDGFR-β^+^ cells prior to this exposure markedly reduces lung myofibroblasts (Supplementary Fig. 7b), thereby indicating the relevance of this pathway to multiple pathogenic processes in the lung.

Possible factors contributing to the reduced bleomycin-induced fibrosis in *Pdgfrb-CreER*^*T2*^*, Klf4*^*(flox/flox)*^ mice could include that *Klf4* deletion in preexisting PDGFR-β^+^ cells reduces proliferation and/or differentiation into myofibroblasts of this lineage. Thus, *Pdgfrb-CreER*^*T2*^*, ROSA26R*^*(YFP/+)*^ mice that also carried *Klf4*^*(flox/flox)*^ or were wild type for *Klf4* were injected with tamoxifen, rested and then bleomycin was administered. Mice were injected with EdU and 12 h later, they were euthanized on day 5 or 14 after bleomycin. Histological analysis of lung sections stained for YFP and EdU shows that *Klf4* deletion does not significantly alter proliferation of cells of the PDGFR-β^+^ cell lineage at day 5 (Supplementary Fig. 11b, c) or 14 (Fig. 3f, g). In addition, the percentage of SMA^+^ myofibroblasts that derive from PDGFR-β^+^ cells is not changed by *Klf4* deletion in PDGFR-β^+^ cells (Fig. 3h, i). Taken together, these findings indicate that, in the context of bleomycin treatment, although *Klf4* deletion in PDGFR-β^+^ cells attenuates lung fibrosis, KLF4 is not required for PDGFR-β^+^ cell proliferation or differentiation into myofibroblasts.

### KLF4 regulates TGFβ signaling and ECM synthesis in PDGFR-β^+^ cells

**Fig. 4:**
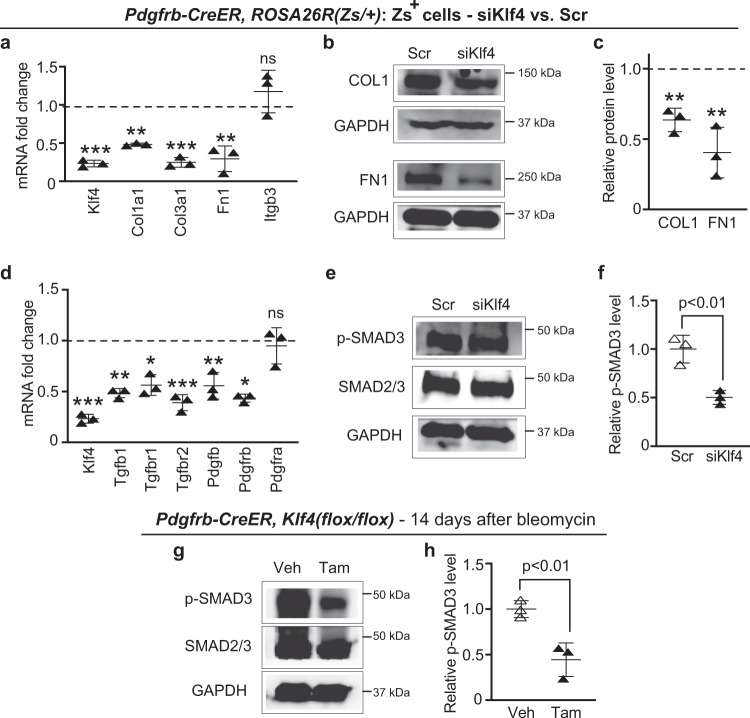
Klf4 reduction in PDGFR-β^+^ cells attenuates levels of TGFβ pathway members and signaling and ECM components. **a–f** Zs^+^ cells were isolated from the lungs of tamoxifen-induced *Pdgfrb-CreER*^*T2*^*, ROSA26R*^*(Zs/+)*^ mice and then subjected to siRNA-mediated knockdown with siKlf4 vs. scrambled (Scr). In **a** and **d**, levels of indicated transcripts relative to Gapdh were determined by qRT-PCR and normalized to Scr. *n* = 3 in triplicate. *, Tgfbr1 (*p* = 0.017), Pdgfrb (*p* = 0.010); **, Col1a1 (*p* = 0.0035), Fn1 (*p* = 0.0042), Tgfb1 (*p* = 0.0034), Pdgfb (*p* = 0.0045); ***, Klf4 (*p* = 0.0006), Col3a1 (*p* = 0.0004), Tgfbr2 (*p* = 0.0007); ns (not significant) vs. Scr. In **b** and **e** and **c** and **f**, western blots for indicated proteins and corresponding densitometry relative to GAPDH and normalized to Scr are shown, respectively. *n* = 3. **, COL1 (*p* = 0.0016), FN1 (*p* = 0.0045) vs. Scr in **b** and *p* = 0.0057 in **f**. **g, h** *Pdgfrb-Cre*^*T2*^, *Klf4*^*(flox/flox)*^ mice were induced with either vehicle (Veh; corn oil) or tamoxifen (Tam), rested, injected orotracheally with bleomycin, and 14 days later, lungs were harvested. Western blots of whole lung lysates for phosphorylated (p)-SMAD3, total SMAD2/3 and GAPDH are shown in **g** with densitometries of p-SMAD3 relative to SMAD2/3 and normalized to Veh (**h**). *n* = 3, *p* = 0.0095. Two-tailed Student’s *t* test was used for all analysis. Data are averages ± SD. Source data are provided as a Source Data file.

To further interrogate mechanisms by which KLF4 in PDGFR-β^+^ cells may induce lung fibrosis, we conducted studies on isolated lung cells. *Pdgfrb-CreER*^*T2*^*, ROSA26R*^*(Zs/+)*^ mice were induced with tamoxifen, and then lung Zs^+^ cells were isolated by FACS and Zs expression was verified in culture (Supplementary Fig. 12). Complementing our findings in mice (Fig. 3b–e), siRNA-mediated Klf4 silencing of these isolated cells reduced mRNA and/or protein levels of key ECM components of the fibrotic response, Col1a1, Col3a1 and Fn1 (Fig. 4a–c). TGFβ-mediated SMAD2/3 phosphorylation is the major pathway implicated in organ fibrosis^41,42^, and the PDGF signaling pathway plays a role as well^43^. Klf4 knockdown in isolated lung Zs^+^ cells from *Pdgfrb-CreER*^*T2*^*, ROSA26R*^*(Zs/+)*^ mice resulted in downregulation of ligands Tgfb1 and Pdgfb and their receptors Tgfbr1, 2 and Pdgfrb, respectively; however, mRNA levels of Pdgfra and Itgb3, known regulators of the ECM and fibrotic response^43,44^, were not altered (Fig. 4a, d). Furthermore, reduction of Klf4 in isolated Zs^+^ cells decreases levels of SMAD3 phosphorylation by ~50% (Fig. 4e, f). To evaluate the in vivo relevance of this finding to fibrotic disease, *Pdgfrb-CreER*^*T2*^*, Klf4*^*(flox/flox)*^ mice were induced with tamoxifen and then exposed to orotracheal bleomycin. Fourteen days later, lung lysates were analyzed by western blot and a similar reduction of phospho-SMAD3 is detected (Fig. 4g, h). Taken together, our data suggest that KLF4 regulates TGFβ-SMAD signaling and thereby ECM synthesis in PDGFR-β^+^ lung cells in culture and in fibrotic lung disease.

### *Klf4* deletion in SMA^+^ cells exacerbates pulmonary fibrosis

**Fig. 5:**
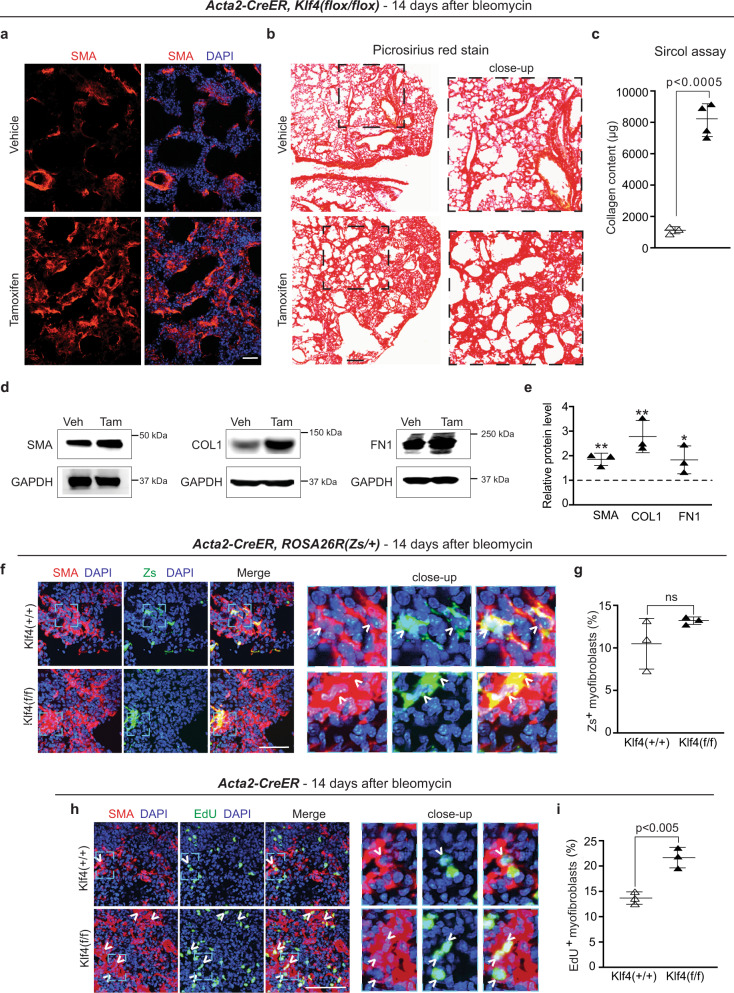
*Klf4* deletion in SMA^+^ cells promotes myofibroblast accumulation and exacerbates bleomycin-induced lung fibrosis. **a–e** *Acta2*-*CreER*^*T2*^, *Klf4*^*(flox/flox)*^ mice were induced with either vehicle (Veh; corn oil) or tamoxifen (Tam), rested and treated with bleomycin. Lungs were analyzed 14 days later. Cryosections were stained for SMA and nuclei (DAPI) in **a** and with Picrosirius red in **b** with dashed boxes shown as close-ups on the right. *n* = 3 mice. Collagen content of the right lung was measured using the Sircol assay (**c**); *n* = 3 mice for vehicle and *n* = 4 mice for tamoxifen inductions in duplicate. *p* = 0.0001. Western blot analysis of lung lysates for SMA, COL1 and FN1 is shown in **d** with densitometric analysis relative to GAPDH and normalized to vehicle (**e**). *n* = 3; *FN1 (*p* = 0.044); **SMA (*p* = 0.0042), COL1 (0.0093) vs. vehicle. **f–i** *Acta2-CreER*^*T2*^ mice carrying *Klf4*^*(flox/flox)*^ or wild type for *Klf4* were induced with tamoxifen, rested and treated with bleomycin, and lungs were analyzed 14 days later. In **f**, mice were also *ROSA26R*^*(Zs/+)*^, and in **h**, mice were injected with EdU 12 h prior to euthanasia. Lung cryosections were stained for SMA and nuclei (DAPI) and either directly imaged for Zs^+^ (**f**) or also stained for EdU (**h**) with boxed regions shown as close-ups on the right. Arrowheads indicate SMA^+^ myofibroblasts that are also Zs (**f**) or EdU^+^ (**h**). The percentage of myofibroblasts that are Zs ^+^ or EdU^+^ was quantified in **g** and **i**, respectively. *n* = 3 mice, four sections per mouse, and on average 132 SMA^+^ myofibroblasts per section. ns, not significant (**g**) and *p* = 0.0042 (**i**). Two-sided Student’s *t* test was used for all analysis. Data are averages ± SD. Source data are provided as a Source Data file. Scale bars, 200 μm (**b**), 50 μm (**a**), 25 μm (**f, h**).

Given that SMA^+^ cells give rise to ~10% of bleomycin-induced lung myofibroblasts (Fig. 1b, c) and that KLF4 plays a prominent role in transformation of PDGFR-β^+^ cells to a profibrotic phenotype (Figs. 3 and 4), we next assessed the role of KLF4 in SMA^+^ cells in fibrogenesis. KLF4 is dynamically upregulated in lung SMA^+^ cells during the early stages of the acute inflammatory phase following bleomycin administration (Supplementary Fig. 13a). To evaluate the effects of deleting *Klf4* in airway and vascular SMCs and the very rare myofibroblasts in the basal lung on subsequent development of fibrosis, *Acta2-CreER*^*T2*^*, Klf4*^*(flox/flox)*^ mice were injected with either tamoxifen or vehicle (corn oil), rested and then treated with a single orotracheal dose of bleomycin. Fourteen days later, mice were euthanized and lungs were processed. Surprisingly, there is a marked increase in lung myofibroblasts with *Klf4* deletion in SMA^+^ cells (Fig. 5a). Furthermore, there is an increase in levels of collagen (Fig. 5b, c) and SMA, COL1 and FN1 proteins (Fig. 5d, e). These findings indicate that, in stark contrast to its role in PDGFR-β^+^ cells (Fig. 3), KLF4 in SMA^+^ cells protects against bleomycin-induced lung fibrosis.

Subsequently, we analyzed the effect of *Klf4* deletion in preexisting SMA^+^ cells on proliferation and fate of this lineage and on myofibroblast proliferation following bleomycin treatment. *Acta2-CreER*^*T2*^*, ROSA26R*^*(Zs/+)*^ mice also carrying *Klf4*^*(flox/flox)*^ or wild type for *Klf4* were induced with tamoxifen, rested, treated with orotracheal bleomycin and then euthanized 14 days later. Twelve hours prior to euthanasia, EdU was administered. Lungs were harvested and stained for SMA, EdU and/or nuclei (DAPI) and directly imaged for Zs. The percentage of Zs^+^ cells that are proliferative (EdU^+^; Supplementary Fig. 13b, c) and the percentage of myofibroblasts that derive from Zs^+^ cells (Fig. 5f, g) are not altered by *Klf4* deletion in preexisting SMA^+^ cells as previously observed with *Klf4* deletion in preexisting PDGFR-β^+^ cells (Fig. 3f–i). Interestingly, the percentage of total myofibroblasts that are proliferative is substantially increased in tamoxifen-induced *Acta2-CreER*^*T2*^*, Klf4*^*(flox/flox)*^ mice (Fig. 5h, i). In sum, these observations indicate that KLF4 in SMA^+^ cells does not cell autonomously regulate proliferation or differentiation to myofibroblasts during bleomycin-induced lung injury, but deletion of *Klf4* in SMA^+^ cells does exacerbate myofibroblast proliferation and accumulation and fibrosis.

### Reduction of KLF4 in SMA^+^ cells induces CCL2 expression

**Fig. 6:**
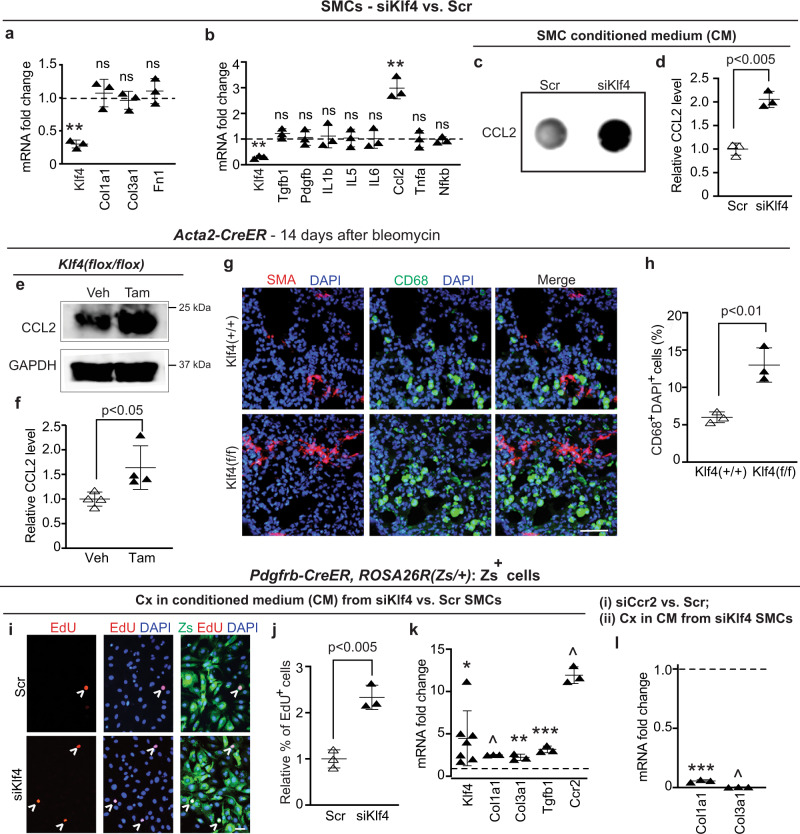
Reduction of Klf4 in SMA^+^ cells increases Ccl2 levels and noncell autonomously induces Ccr2-mediated collagen expression in PDGFR-β^+^ cells. **a**–**d** Klf4 siRNA or scrambled (Scr) RNA treatment of mouse airway SMCs. In **a** and **b**, transcript levels were assayed by qRT-PCR. *n* = 3 in triplicate. **, Klf4 (*p* = 0.0035), Ccl2 (*p* = 0.0013), ns (not significant) vs. Scr. Dot blots show CCL2 protein levels in conditioned medium (CM) (**c**) with densitometry normalized to Scr (**d**). *n* = 3, *p* = 0.0010. **e**–**h** The lungs of *Acta2-CreER*^*T2*^ mice carrying *Klf4*^*(flox/flox)*^ or *Klf4*^*(+/+)*^ were analyzed 14 days after a bleomycin dose. In **e** and **f**, mice were induced with vehicle (Veh; corn oil) or tamoxifen (Tam) prior to bleomycin, and lung lysates were subjected to western for CCL2 and GAPDH with densitometry relative to GAPDH and normalized to vehicle. *n* = 4, *p* = 0.033. In **g** and **h**, mice were induced with tamoxifen prior to bleomycin, and lung sections were stained for SMA and macrophage marker CD68. Percent of DAPI^+^ cells that express CD68 was quantified (**h**). *n* = 3 mice, five sections per mice and an average of 906 DAPI^+^ cells per section, *p* = 0.0073. **i**–**l** *Pdgfrb-CreER*^*T2*^*, ROSA26R*^*(Zs/+)*^ mice were induced with tamoxifen, and Zs^+^ cells were isolated by FACS. In **i**–**k**, Zs^+^ cells were cultured (cx) in CM from airway SMCs pretreated with siKlf4 or Scr RNA. In **i**, cells were cultured with EdU for 8 h prior to staining for EdU and nuclei (DAPI) and direct Zs imaging. The percent of DAPI^+^ cells that were EdU^+^ (arrowheads) was quantified and normalized to percentage for Scr CM-treated cells (**j**). *n* = 3 experiments, five microscopic fields of view per experiment and on average 279 DAPI^+^ cells per field, *p* = 0.0022. In **k**, transcript levels relative to Gapdh were measured by qRT-PCR and normalized to Scr CM-treated cells. *n* = 7 for Klf4 and *n* = 3 for other transcripts. *p = 0.016; **p < 0.0043; ***p = 0.0008; ^, <0.0001 vs. Scr. In **l**, Zs^+^ cells were subjected to siCcr2 vs. Scr and then cultured in medium preconditioned by siKlf4-treated SMCs. qRT-PCR was used to measure Col1a1 and Col3a1 mRNA levels. *n* = 3, ***p = 0.0008, ^*p* < 0.0001 vs. Scr. Two-tailed Student’s *t* test was used for all analysis. Data are averages ± SD. Source data are provided as a Source Data file. Scale bars, 50 μm.

To further investigate mechanisms underlying the protective role of KLF4 in SMA^+^ cells during lung fibrosis, we evaluated the effects of siRNA-mediated Klf4 knockdown in murine airway SMCs. In contrast to the results with PDGFR-β^+^ cells (Fig. 4), Klf4 silencing in SMCs does not alter levels of ECM components Col1a1, Col3a1 or Fn1 (Fig. 6a) suggesting that *Klf4* deletion in SMCs may not directly influence ECM deposition by SMCs during fibrosis. Thus, we explored potential noncell autonomous effects, starting with the expression of select growth factors, cytokines, chemokines previously implicated in fibrosis^45,46^. Transcript levels of Tgfb1, Pdgfb, Tnfa, interleukins 1b, 5 and 6 and the relevant TF Nfkb do not change with Klf4 knockdown in SMCs (Fig. 6b), whereas mRNA and secreted protein levels of CCL2 (also known as monocyte chemoattractant protein-1) are markedly increased (Fig. 6c, d). In notable contrast, Klf4 knockdown in PDGFR-β^+^ cells does not alter Ccl2 expression (Supplementary Fig. 14a). CCL2 is a chemokine for myeloid cells, and treatment with anti-CCL2 antiserum or gene transfer of a dominant negative Ccl2 attenuates lung fibrosis in mice^47–49^. As a validation of the in vivo relevance of our findings showing KLF4 regulation of Ccl2 in cultured SMCs, deletion of *Klf4* in SMA^+^ cells increases both CCL2 in lung lysates and the number of lung cells expressing the macrophage marker CD68 in bleomycin-treated mice (Fig. 6e–h). Together, these findings suggest *Klf4* deletion in SMA^+^ cells worsens bleomycin-induced lung fibrosis at least partly by increasing secretion of CCL2.

### Klf4 silenced SMCs induce fibrotic changes in PDGFR-β^+^ cells via CCR2

In addition to promoting macrophage accumulation, we queried whether conditioned medium from siKLF4-treated SMCs induces profibrotic transformation of PDGFR-β^+^ lung cells via the CCL2 receptor CCR2. To this end, Zs^+^ cells were isolated by FACS from lungs of tamoxifen-induced *Pdgfrb-CreER*^*T2*^*, ROSA26R*^*(Zs/+)*^ mice. These Zs^+^ cells were cultured in conditioned medium from siKlf4-treated SMCs (experimental medium) or from scrambled (Scr) siRNA-treated SMCs (control medium) for 24 h. Zs^+^ cells cultured in experimental medium are ~2.5 more proliferative as determined by EdU incorporation (Fig. 6i, j). Moreover, experimental medium induces these cells to express increased levels of Klf4, Col1a1, Col3a1 and Tgfb1 (Fig. 6k), consistent with previous findings that KLF4 mediates expression of collagens and Tgfb1 in PDGFR-β^+^ cells (Figs. 3b–e and 4a–c). Furthermore, Ccr2 transcript levels are elevated by ~12-fold in Zs^+^ cells cultured in experimental as opposed to control medium (Fig. 6k), and thus, we evaluated whether collagen levels are regulated by the CCL2-CCR2 axis in these cells. Zs^+^ cells were exposed to Scr RNA or siRNA against Ccr2 and then incubated in experimental conditioned medium. Ccr2 knockdown in Zs^+^ cells downregulates Col1a1 and Col3a1 transcripts by more than 90-fold with culturing in experimental conditioned medium (Fig. 6l) but has no effect with culturing in standard medium (low glucose DMEM with supplements, see “Methods”; Supplementary Fig. 14b). Collectively, these data suggest that CCL2 secreted by siKlf4-treated SMCs signal through the CCR2 receptor on PDGFR-β^+^ cells to induce collagen expression.

### KLF4 differentially regulates *Ccl2* and *Tgfb1* in SMCs and PDGFR-β^+^ cells

**Fig. 7:**
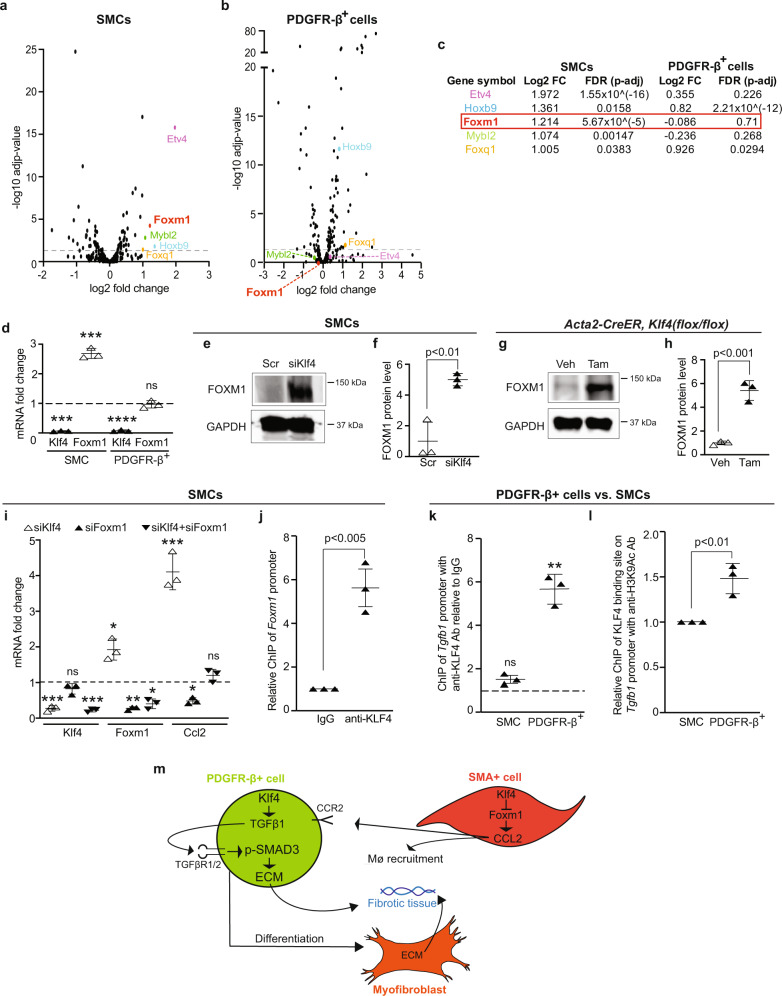
KLF4 differentially regulates downstream genes in SMCs and PDGFR-β^+^ cells. Lung PDGFR-β^+^ cells (Zs^+^ cells from *Pdgfrb-CreER*^*T2*^*, ROSA26R*^*(Zs/+)*^ mice) and SMCs were compared. **a**–**f** Cells were treated with Klf4 siRNA or Scr RNA. In **a**–**c**, cells were subjected to bulk RNA-seq; *n* = 3. Of the TFs predicted with TRANSFAC to bind *Ccl2* promoter (see Supplementary Data 3), expression levels of genes present in RNA-seq data are shown (**a**, **b**). Among predicted *Ccl2*-binding genes, the five most upregulated genes in SMCs are indicated, and log2 fold change (FC) and false discovery rate (FDR) in SMCs and PDGFR-β^+^ cells are displayed (**c**). In **d**, qRT-PCR was used to assess Klf4 and Foxm1 transcript levels relative to Gapdh and normalized to Scr; *n* = 3 in triplicate. ***, Klf4_SMC (*p* = 0.0004), Foxm1_SMC (*p* = 0.0006), Klf4_PDGFR-β^+^ (*p* < 0.0001) and ns (not significant) with siKlf4 vs. Scr. In **e**, **f**, westerns for FOXM1 and GAPDH with densitometry of their ratio and normalized to Scr; *n* = 3, *p* = 0.0060. **g**, **h** *Acta2-CreER*^*T2*^*, Klf4*^*(flox/flox)*^ mice were induced with vehicle (corn oil) or tamoxifen, and lung lysates were evaluated as in **e** and **f** with normalization to vehicle; *n* = 3, *p* = 0.0009. **i** SMCs were treated with Scr, siKlf4, siFoxm1 or both siKlf4 and siFoxm1. Transcript levels of Klf4, Foxm1 and Ccl2 relative to Gapdh as assessed by qRT-PCR and normalized to Scr; *n* = 3. *, siKlf4_Foxm1 (*p* = 0.012), si-both_Foxm1 (*p* = 0.012), siFoxm1_Ccl2 (*p* = 0.023); **, siFoxm1_Foxm1 (*p* = 0.0036); ***, siKlf4_Klf4 (*p* = 0.0003), si-both_Klf4 (*p* < 0.0001), siKlf4_Ccl2 (*p* = 0.0007); ns vs. Scr. **j** ChIP assay for SMCs using anti-KLF4 antibody or control IgG, followed by qPCR with primers for *Foxm1* promoter. Signal for coprecipitated DNA with anti-KLF4 antibody normalized to IgG is shown; *n* = 3, *p* = 0.0021. **k**, **l** ChIP was performed in SMCs and PDGFR-β^+^ cells using qPCR primers for *Tgfb1* promoter. In **k**, anti-KLF4 antibody or IgG was used for pulldown and coprecipitated DNA with anti-KLF4 antibody is relative to IgG. *n* = 3, SMC (ns) and PDGFR-β^+^ (*p* = 0.0071) with anti-KLF4 antibody vs. IgG. In **l**, lysates were subjected to immunoprecipitation with anti-H3K9Ac antibody and amount of coprecipitated DNA from each cell type is relative to PCR product amplified from input samples reserved before immunoprecipitation. This ratio for PDGFR-β^+^ cells is normalized to SMCs; *n* = 3, *p* = 0.0076. Two-tailed Student’s *t* test was used for analysis (**d**, **f**, **h**–**l**). **m** Mesenchymal cell-type-specific KLF4 effects in lung fibrosis. Data are averages ± SD. Source data are provided as a Source Data file.

To gain further mechanistic insights into KLF4-mediated differential regulation of downstream targets in SMCs and PDGFR-β^+^ cells, these cells were treated with Scr RNA or siRNA targeting Klf4 and then subjected to bulk RNA-seq (Supplementary Data 1 and 2). Consistent with qRT-PCR and western/dot blot analyses (see Fig. 6b–f and Supplementary Fig. 14a), Ccl2 levels were increased (~5.7-fold, padj < 0.005) with knockdown of Klf4 in SMCs whereas in PDGFR-β^+^ cells, siKlf4 treatment did not alter Ccl2 levels. We next sought TFs that bind *Ccl2* *cis*-regulatory regions, induce expression and are differentially expressed with Klf4 knockdown, in a similar manner to Ccl2. To this end, using the TRANSFAC database, 407 TFs were identified that are predicted to bind between −5000 bp to +100 bp (transcription start site of *Ccl2* is at position +1) (Supplementary Data 3). Using ingenuity pathway analysis, this data set was aligned to the RNA-seq data, yielding expression data for 234 and 230 TFs in SMCs and PDGFR-β^+^ cells, respectively, that are predicted to bind *Ccl2*, and Volcano plots were generated (Fig. 7a, b). Of the five most highly upregulated transcripts in SMCs, three of these genes, ETS variant TF 4 (Etv4), Foxm1 and MYB proto-oncogene like 2 (Mybl2), are not significantly upregulated in PDGFR-β^+^ cells, but there is a trend to an increased Etv4 level in PDGFR-β^+^ cells (Fig. 7c).

We elected to further investigate Foxm1 based on these findings and recent studies demonstrating that KLF4 negatively regulates Foxm1 expression in gastrointestinal cancer cell lines, FOXM1 positively regulates Ccl2 expression in hepatocytes and *Foxm1* deletion in COL1a2^+^ cells attenuates bleomycin-induced lung fibrosis^50–52^. As with RNA-seq, using qRT-PCR, we found that siRNA targeting Klf4 increases Foxm1 transcript levels in SMCs but not in PDGFR-β^+^ cells (Fig. 7d). FOXM1 protein is increased in SMCs by 5.0 ± 0.4-fold with siKlf4 treatment and in lung lysates by 5.4 ± 0.8-fold with tamoxifen-treatment of *Acta2-CreER, Klf4*^*(flox/flox)*^ mice (Fig. 7e–h). Importantly, concomitant knockdown of Klf4 and Foxm1 in SMCs prevents the robust upregulation of Ccl2 observed with only Klf4 silencing (Fig. 7i). Chromatin immunoprecipitation (ChIP) assay with an anti-KLF4 or isotype control antibody suggests that KLF4 robustly binds the promoter region of *Foxm1* (Fig. 7j). In addition, we found that pyruvate dehydrogenase kinase 1, which is implicated as a positive regulator of Foxm1 mRNA levels in a human lung fibroblast cell line^51^, is upregulated in SMCs but not PDGFR-β^+^ cells with Klf4 knockdown (Supplementary Fig. 15).

As with the Foxm1-Ccl2 axis, upon Klf4 knockdown, Tgfb1 levels are differentially altered in lung PDGFR-β^+^ cells (downregulated; Fig. 4d) and SMCs (unchanged; Fig. 6b). It was previously reported that in vimentin^+^ noncardiomyocyte cells isolated from the neonatal mouse heart, KLF4 binds the Tgfb1 promoter and induces transcription^29^. We performed the ChIP assay using an anti-KLF4 or isotype control antibody and found that KLF4 binding to the *Tgfb1* promoter region was increased in PDGFR-β^+^ cells (5.7 ± 0.7-fold) but not in SMCs (Fig. 7k). Next, we queried whether this differential KLF4 binding is due to differences in chromatin accessibility which would facilitate interactions with TFs. Indeed, ChIP of the Tgfb1 promoter with an antibody targeting acetylated histone H3 lysine9 (H3K9Ac), which marks open chromatin, demonstrates 50% higher signal in PDGFR-β^+^ cells than SMCs (Fig. 7l). Taken together, our data indicate that KLF4 differentially regulates profibrotic mediators in distinct lung cell types.

## Discussion

In the healthy lung, epithelial cells lining the airways and alveoli are a critical line of defense against toxins. Chronic insults to these cells can trigger the fibrotic response largely via deleterious effects on underlying mesenchymal cells. During the pathogenesis of lung fibrosis, mesenchymal cells are implicated as the major source of excessive myofibroblasts and ECM components. Although aberrant signaling from epithelium to mesenchyme is considered a key element of the pathogenesis of lung fibrosis, the role of signaling between different mesenchymal cell types is not well delineated. Herein, using bleomycin-induced lung fibrosis, we demonstrate that PDGFR-β^+^ cells are the major source of myofibroblasts (Fig. 1), and KLF4 in PDGFR-β^+^-derived cells is critical for the accumulation of excess myofibroblasts and ECM (Figs. 2–4). In stark contrast, KLF4 in SMA^+^ cells is protective, and reduction of Klf4 in these cells enhances the number of lung myofibroblasts and noncell autonomously induces PDGFR-β^+^ cells to generate excess ECM components (Figs. 5 and 6). Mechanisms underlying cell-type-specific effects of KLF4 in SMCs and PDGFR-β^+^ cells are evaluated (Fig. 7) and summarized in Fig. 7m.

Myofibroblasts are classically considered a key cell type in the pathobiology of lung fibrosis—important for lung contraction and accumulation of excess ECM—but their cellular origins are incompletely defined. Fate mapping studies suggest that diverse mesenchymal cell types give rise to a substantial portion of pathological myofibroblasts whereas it is controversial whether epithelial cells contribute^13–17,41,53^. These source mesenchymal cell populations include ADRP^+^ lipofibroblasts, FoxD1^+^ progenitor-derived pericytes, Gli1^+^ MSC-like cells and Axin2^+^ myofibrogenic progenitors and the latter three cell populations—pericytes, MSC-like cells and Axin2^+^ progenitors—express PDGFR-β. Following a single bleomycin dose, fate mapping of Gli1^+^ cells suggests that Gli1^+^PDGFR-β^+^ MSC-like cells give rise to 37% of lung myofibroblasts at 14 day^15^ whereas we find that PDGFR-β^+^ cells give rise to ~85% of myofibroblasts at day 7 and ~70% of myofibroblasts at day 14 (Fig. 1). Thus, both Gli1^+^PDGFR-β^+^ and Gli1^-^PDGFR-β^+^ cell populations are likely important sources of pathological myofibroblasts. Albeit to a considerably lesser extent than PDGFR-β^+^ cells, recent studies suggest that PDGFR-α^+^ cells contribute^14,54^. One of these studies, by Zepp et al., also implicates peribronchiolar Axin2^+^PDGFR-α^-^PDGFR-β^+^ cells that have enriched RNA levels of Acta2, transgelin and desmin as an important source of bleomycin-induced myofibroblasts^14^. Interestingly, immunohistochemistry reveals that less than 10% of Axin2^+^ cells surrounding airways or blood vessels express SMA or SM22α, and thus, the authors suggest that the Axin2^+^ myofibrogenic cells may be primed for, but are not committed to, SMC or myofibroblast lineages^14^. In light of these prior findings, it is notable that a study by El Agha et al. suggests that SMA^+^ cells are not a source of bleomycin-induced lung myofibroblasts^13^, and our fate mapping results indicate that only ~10% of lung myofibroblasts derive from preexisting SMA^+^ cells (Fig. 1). Thus, Acta2-CreER may not efficiently mark the Axin2^+^ myofibrogenic progenitor cell population and/or Axin2^+^Acta2^-^ cells may be the primary Axin2^+^ myofibrogenic progenitors.

The potential significance of the ~15% decrease in PDGFR-β-derived myofibroblasts during the second week after bleomycin exposure in our studies is not delineated nor is the underlying mechanism. Possible mechanisms include an increased contribution of another source during this time period or apoptosis or SMA downregulation in the PDGFR-β^+^ cell lineage. Our multicolor clonal analysis suggests the large contribution of PDGFR-β^+^ cells to the pathological myofibroblast pool is through clonal expansion of PDGFR-β^+^ cells with limited mixing of clones. This result is similar to what has been shown with the lineage deriving from cells that express the mesenchyme marker TBX4^55^.

Mesenchymal cells change profoundly during the pathogenesis of lung fibrosis, and our results indicate that KLF4, a key regulator of cell transitions, is profibrotic in PDGFR-β^+^ cells and, through noncell autonomous effects, is antifibrotic in SMA^+^ cells (Figs. 3 and 5). Such cell-type-specific effects of KLF4 likely underlie a large part of the disparate effects of KLF4 in renal fibrosis^30^. Interestingly, in the carotid artery adventitia, deletion of *Klf4* in SMC-derived SCA1^+^SMC marker^-^ progenitors results in elevated collagen levels and expansion of this vascular layer^56^. In the lung, KLF4 is implicated in the normal perinatal accumulation of myofibroblasts as *Klf4*^*(−/−)*^ neonates lack alveolar myofibroblasts, and expression of KLF4 in lung fibroblasts isolated from *Klf4* null mice induces expression of SMA, collagen and fibronectin^23^. In contrast, KLF4 overexpression either in vivo (transgenic mice) in unspecified cell types reduces bleomycin-induced lung fibrosis or in cultured human alveolar epithelial cells attenuates TGFβ1-induced downregulation of the epithelial marker E-cadherin and upregulation of fibronectin^33^.

Our studies indicate that upon bleomycin treatment, KLF4 expression is enhanced in PDGFR-β^+^ cells and regions of the lung adjacent to SMA^+^ fibrotic patches, but in agreement with a recent paper^33^, this expression is markedly reduced within the SMA^+^ fibrotic patches of the human IPF or murine lung (Fig. 2). These findings suggest that KLF4 may be important in the initial phases of fibrosis but superfluous once fibrotic patches are established. *Klf4* deletion in PDGFR-β^+^ cells attenuates bleomycin-induced accumulation of myofibroblasts and lung fibrosis (Fig. 3). In a distinct model of lung injury, we recently found that *Klf4* deletion in PDGFR-β^+^ cells prevents hypoxia-induced expansion of vascular SMCs and pulmonary hypertension^57^, and herein, similar to our results with bleomycin, we observed that deletion of *Klf4* in PDGFR-β^+^ cells markedly reduces hypoxia-induced lung myofibroblasts (Supplementary Fig. 7). Surprisingly, with bleomycin treatment, we did not detect an effect of *Klf4* deletion in PDGFR-β^+^ cells on proliferation and percent contribution to SMA^+^ myofibroblasts even though there is an overall reduction in myofibroblasts and SMA protein levels. Bleomycin treatment of mice stimulates lung PDGFR-β^+^ cells to express collagen, and Klf4 knockdown in PDGFR-β^+^ cells reduces ECM component levels (Figs. 1 and 4). Thus, we suggest that in PDGFR-β^+^ cells, bleomycin-induced KLF4 cell autonomously enhances ECM production and speculate that the resulting increased stiffness of the lung parenchyma contributes to the conversion of select populations of both PDGFR-β^+^ and PDGFR-β^-^ cells to SMA^+^ myofibroblasts. This hypothesis is consistent with the recent finding that human placental pericytes upregulate SMA expression with culturing on stiff polyacrylamide hydrogels^58^.

The TGFβ pathway is widely implicated in the pathogenesis of tissue fibrosis^41^—even referred to as the master regulator of fibrosis by some investigators^42^—and our findings indicate that KLF4 induces TGFβ signaling during this process in the lung. We previously demonstrated a key role of the TGFβ pathway in PDGFR-β^+^ pericytes for embryonic brain morphogenesis^59^, and herein, *Klf4* deletion in PDGFR-β^+^ cells prior to bleomycin administration is shown to downregulate phosphorylation of the TGFβ effector SMAD3 in the lung (Fig. 4). In addition, Klf4 knockdown in isolated PDGFR-β^+^ cells reduces levels of TGFβ pathway members—including the ligand Tgfb1, receptors Tgfbr1/r2 and phospho-SMAD3—and collagens 1a1, 3a1 and fibronectin (Fig. 4). In complementary experiments with vimentin^+^ noncardiomyocyte cells of the neonatal mouse heart, KLF4 has been shown to activate the TGFβ1 promoter and enhance both SMA levels and angiotensin II-induced expression of Col1a1 and Col3a1^29^. In contrast, a prior study of cells isolated from the rodent lung by enzymatic digestion and cultured under conditions resulting in fibroblast-like morphology reported that KLF4 binds SMAD3 and thereby, inhibits activation of the *Acta2* promoter^31,32^. Importantly, latent TGFβ is activated by many heterodimeric integrins that contain the integrin αv subunit, and *Pdgfrb-Cre, Itgav*^*(flox/flox)*^ mice are protected against bleomycin-induced lung fibrosis^36^. Future investigations into potential interactions of KLF4 and regulatory elements of the integrin αv gene in lung mesenchymal cells are likely to be of high yield.

In contrast to our results with PDGFR-β^+^ cells, KLF4 reduction in SMA^+^ cells of the normal lung, which are predominantly airway and vascular SMCs, exacerbates fibrosis and myofibroblast accumulation in bleomycin-treated mice, but in culture has no effect on levels of Tgfb1, collagens 1a1, 3a1 and fibronectin (Figs. 5 and 6). Yet, this perturbation markedly induces CCL2 levels and accumulation of CD68^+^ cells, and CCL2 upregulation has previously been demonstrated in lungs of IPF patients and bleomycin-treated rodents^48,60^. Of note, there is precedence for SMCs secreting chemokines and cytokines during the inflammatory response^61,62^. We found that conditioned medium from SMCs subjected to Klf4 knockdown induces PDGFR-β^+^ cells to proliferate and express increased levels of Klf4, Ccr2, Tgfb1 and collagens 1a1 and 3a1, and the collagen upregulation is Ccr2 dependent. In the context of bleomycin administration, lung hydroxyproline content is reduced by global *Ccr2* deletion or gene transfer of a dominant negative *Ccl2*^48,63^. A trial of carlumab, an inhibitor that binds and neutralizes profibrotic activities of CCL2, unfortunately showed no benefit in IPF patients and in fact a trend to worse outcomes^64^. Surprisingly, however, carlumab treatment was associated with an increase—not the expected decrease—in free CCL2 serum levels^64^, suggesting that alternate means to reduce CCL2-CCR2 signaling remains a potentially viable therapeutic approach.

Thus, a relevant mechanistic question arises: how does KLF4 induce markedly distinct effects in PDGFR-β^+^ cells and SMCs? Among TFs bioinformatically predicted to bind within the 5-kb region upstream of *Ccl2*, our unbiased transcriptomic screen of Klf4 silenced cells identified Foxm1 as one of the few most upregulated genes in SMCs that is not also upregulated in PDGFR-β^+^ cells (Fig. 7). Klf4 has been shown to negatively regulate Foxm1 in a gastrointestinal cancer cell line, and FOXM1 induces CCL2 levels in hepatocytes^50,52^. Our studies demonstrate a link in lung SMCs between Klf4 downregulation to upregulation of FOXM1 and in turn, increased CCL2. Interestingly, no such Klf4-mediated regulation of Foxm1 or Ccl2 was observed in PDGFR-β^+^ cells. In contrast, Klf4 silencing results in Tgfb1 levels that are increased in PDGFR-β^+^ cells but unaltered in SMCs. These differential effects are likely due to the increased accessibility (as indicated by histone H3K9 acetylation) and hence binding of KLF4 to the *Tgfb1* promoter in PDGFR-β^+^ cells.

Taken together, our results indicate that KLF4 is a major player in the lung mesenchyme during fibrosis, having opposing cell-type-specific effects: profibrotic in PDGFR-β^+^ cells and antifibrotic in SMA^+^ cells. This work also highlights the importance of studying intercellular signaling between mesenchymal cells and their transitions during the pathogenesis of lung fibrosis. Further studies of the role of diverse mesenchymal cell types in the course of lung fibrosis are warranted and promise to advance novel therapeutic strategies for this lethal disease.

## Methods

### Animals

C57BL/6 wild-type mice and Cre reporter *ROSA26R*^*(ZsGreen1/ZsGreen1)*^, *ROSA26R*^*(mTmG/mTmG)*^ and *ROSA26R*^*(YFP/YFP)*^ mice were obtained from Jackson Laboratory^65–67^, and *Klf4*^*(flox/flox)*^ mice were from purchased from the Mutant Mouse Resource & Research Center^68^. *Acta2-CreER*^*T2*^, *Pdgfrb-CreER*^*T2*^ and the multicolor *ROSA26R*^*(Rb/Rb)*^ mice were also used^24,39,69,70^. Experiments included male and female mice aged 3–4 months and sex and age-matched controls. Mice are housed in rooms with a 12 h light/12 h dark cycle, ambient temperature of 69–71 °F and 38–50% humidity.

### Tamoxifen, bleomycin and hypoxia treatment

For fate mapping, clonal analysis and cell isolation, mice were injected intraperitoneally with tamoxifen (1 mg or ~40 mg/kg body weight per day for 5 days). For in vivo *Klf4* deletion, mice were injected with tamoxifen (1 mg or ~40 mg/kg per day for 10 days) or with equivalent volume of corn oil (vehicle). After tamoxifen injections, mice were rested for 5 days before bleomycin (Sigma-Aldrich) or hypoxia treatment. Bleomycin was administered orotracheally as a single dose of 1.5 U/kg body weight. As a control for bleomycin treatment, a similar volume of PBS was given orotracheally. Mice were euthanized and lungs were harvested 5, 7, 14 or 21 days after bleomycin or PBS administration. Alternatively, mice were exposed to normoxia or hypoxia (FiO_2_ 10%) for 21 days in a rodent hypoxia chamber with a calibrated oxygen controller and sensor (BioSpherix) and then euthanized, and lungs were harvested.

### Lung preparation and immunohistochemistry

To prepare for sectioning and cell isolation, after euthanizing mice, the pulmonary vasculature was flushed by injecting PBS through the right ventricle. For vibratome sections, lungs were inflated by tracheal injection of 2% low-melting agarose through the trachea. Lungs were harvested, incubated in Dent’s fixative (4:1 methanol/dimethyl sulfoxide) overnight at 4 °C and then stored in 100% methanol at −20 °C. In preparation for staining, lungs were bleached in 5% H_2_O_2_ in methanol, rehydrated sequentially in 75%, 50% and 25% and 0% methanol in PBS. A vibratome was used to cut 150  μm thick sections. For cryosections, flushed lungs were harvested and fixed in 4% paraformaldehyde (PFA) overnight, washed and incubated in 30% sucrose. Lungs were then embedded in optical cutting temperature compound (Tissue-Tek), frozen and stored at −20 or −80 °C. A cryostat was used to cut 10–30 µm thick sections. Both vibratome sections and cryosections were blocked in 0.1% Triton X-100 in PBS (PBS-T) containing 5% goat serum, washed with PBS-T and incubated with primary antibodies overnight at 4 °C. On the next day, sections were washed with PBS-T, incubated in secondary antibodies for 2 h, washed again with PBS-T and mounted in fluorescence mounting medium (DAKO).

Lungs from human adult IPF patients and age-matched controls were fixed in formalin, paraffin embedded and sectioned. Sections were deparaffinized with xylene and rehydrated into water through serial dilutions of ethanol. Rehydrated sections were washed with TNT (10 mM Tris-HCl, pH 8.0, 150 mM NaCl, 0.2% Tween-20) for 10 min. Sections were then incubated in prewarmed (65 °C) antigen retrieval buffer for 30 min in a microwave per manufacturer’s instructions (DAKO). After cooling to room temperature, sections were washed in TNT buffer for 10 min, blocked in PBS-T containing 5% goat serum for 30 min and immunostained as described above for mouse sections.

Primary antibodies used were chicken anti-GFP (1:100, Abcam), rabbit anti-KLF4 (1:100-200, Cell Signaling), rat anti-CD68 (1:200, Bio-Rad), rat anti-MECA32 (1:15, Developmental Studies Hybridoma Bank), directly conjugated Cy3 anti-SMA (1:150-250, Sigma-Aldrich), and biotinylated anti-PDGFR-β (1:50, R&D). Elite ABC reagents (Vector Laboratories) and fluorescein tyramide system (PerkinElmer) were used to amplify the biotinylated PDGFR-β staining^24^. Secondary antibodies (1:250–500) were raised in goat and conjugated to either Alexa-488 (anti-chicken [Abcam], anti-rabbit [Invitrogen]), −564, (anti-rabbit, anti-rat [Invitrogen]), or −647 fluorophore (anti-rabbit [Invitrogen]). Nuclei were visualized by DAPI (1:1000, Sigma-Aldrich).

In the bleomycin model, lung fibrosis is patchy and thus, most sections include both highly fibrotic and non/low fibrotic areas. Fibrotic lung regions were considered SMA+ areas in the parenchyma that were distant from airways or blood vessels. These regions were generally hypercellular (as evidenced by staining with DAPI) and had distorted alveolar structures. In contrast, nonfibrotic regions had relatively intact alveoli, little or no SMA+ cell accumulation in the parenchyma (beyond airway or vascular SMCs) and were not hypercellular.

### Lung collagen content

For qualitative analysis of collagen content, the Picrosirius Red Stain Kit was used per the instructions of the manufacturer (Abcam). Lung cryosections (10 µm) were hydrated in distilled water, incubated in Picrosirius red solution for 1 h, rinsed twice in acetic acid solution, dehydrated in absolute alcohol and mounted in DPX mounting medium (Sigma-Aldrich). For quantitative analysis, Sircol Collagen Assay was performed according to the instructions and with buffers supplied by the manufacturer (Biocolor). Briefly, the entire right lung was disrupted using TissueLyser II (Qiagen), digested overnight in pepsin and acetic acid and centrifuged at 18,400 × g for 30 min. The supernatant was collected, and 1 ml Sircol dye was incubated with 100 μl of the supernatant for 1 h. This sample was centrifuged at 13,500 × g for 10 min, and the resulting pellet was washed in the acid salt wash buffer and resuspended in 250 μl alkali buffer. Optical density (OD) at 555 nm was measured, and the collagen concentration was determined by comparing the OD of samples with that of standards of known collagen concentrations.

### Fate mapping

Mice carrying an inducible Cre recombinase (*Pdgfrb*-*CreER*^*T2*^ or *Acta2*-*CreER*^*T2*^) and a Cre reporter [*ROSA26R*^(*mTmG*/+)^, *ROSA26R*^(*Zs*/+)^ or *ROSA26R*^(*YFP*/+)^] were injected with tamoxifen, rested and a single orotracheal dose of PBS (control group) or bleomycin (experimental group) was administered. On day 7 or 14 after orotracheal treatment, mice were euthanized and lungs were harvested. Lung sections were stained for SMA and nuclei (DAPI) as well as with an antibody directed against GFP or directly imaged for Zs. The contribution of different lineages to the myofibroblast pool were determined by scoring the percentage of parenchymal SMA^+^ DAPI^+^ cells (excluding vascular and airway SMCs) that were also marked by the Cre reporter.

### Clonal analysis

*Pdgfrb*-*CreER*^*T2*^*, ROSA26R*^(*Rb*/+)^ mice were injected with tamoxifen, rested and then exposed to a single orotracheal dose of PBS or bleomycin. Fourteen days later, lungs were harvested. Cryosections were stained with DAPI, and Rb colors (i.e., Cerulean, mOrange, and mCherry) were directly imaged. The number of contiguous cells of the same Rb color was scored to determine the clonal patch size. Patch area of congruent cells marked with the same fluorophore were determined using Image J software.

### Cell proliferation in the murine lung

*Pdgfrb-CreER*^*T2*^*, ROSA26R*^(*YFP*/+)^ mice that were also either *Klf4*^*(flox/flox)*^ or wild type for *Klf4* were injected with tamoxifen, rested, subjected to orotracheal bleomycin and euthanized 5 or 14 days thereafter. Twelve hours prior to euthanasia, mice were injected intraperitoneally with 2.5 mg of EdU (Thermo Fisher Scientific). Harvested lungs were fixed in 4% PFA overnight, permeabilized in 0.5% PBS-T for 30 min, stained with the Click-iT EdU Alexa Fluor 555 Imaging Kit per instructions of the manufacturer (Thermo Fisher Scientific) and then costained for YFP and nuclei (DAPI). Per each genotype analyzed, the percent of YFP^+^ cells that express EdU was determined.

### Isolation of lung PDGFR-β^+^ and SMA^+^ cells

Lungs were finely minced and incubated in 2 mg/ml collagenase Ι (Worthington) in PBS at 37 °C for 20 min. The digestion mixture was passed through a 14-gauge pipetting needle (Cadence Science), incubated at 37 °C for an additional 20 min and then filtered through a 70 μm cell strainer (Falcon). This sc suspension filtrate was centrifuged at 340 × *g* for 5 min, and the pellet was resuspended in cold PBS with 1% fetal bovine serum (FBS; Invitrogen). The resuspended cells were incubated with either PE-conjugated anti-PDGFR-β antibody (1:200, Miltenyi Biotec) for 20 min in the case of *Pdgfrb-CreER*^*T2*^, *Klf4*^*(flox/flox)*^ mice or DAPI (1:1000) to label dead cells for 10 min in the case of *Pdgfrb*-*CreER*^*T2*^, *ROSA26R*^(*Zs*/+)^ mice. The sample was then washed in cold PBS with 1% FBS and centrifuged at 340 × *g* for 5 min. The resulting pellet was resuspended in PBS containing 1% FBS and 0.02% EDTA. DAPI (1:2000) was added to cells from *Acta2*-*CreER*^*T2*^, *ROSA26R*^(*Zs*/+)^ mice or cells from *Pdgfrb-CreER*^*T2*^, *Klf4*^*(flox/flox)*^ mice that had undergone incubation with anti-PDGFR-β antibody. Finally, Zs^+^DAPI^−^ or PDGFR-β^+^DAPI^−^ cells were sorted on a BD FACSAria II cell sorter. Cells stained with DAPI alone were used as a control for anti-PDGFR-β antibody specificity. Lungs from C57BL/6 mice were processed similarly to those of *Pdgfrb*-*CreER*^*T2*^, *ROSA26R*^(*Zs*/+)^ mice, and isolated cells were used as a control for auto-fluorescence in the Zs channel. FlowJo was used to analyze data.

### Western blot

Lung tissues or cells were lysed in RIPA buffer containing complete protease inhibitor and PhosSTOP phosphatase inhibitor cocktails (Roche Applied Science). Lysate was centrifuged at 13,500 × *g* for 10 min at 4 °C, and the supernatant was collected. Protein concentrations were determined by the BCA assay (Pierce). Laemmli buffer (Bio-Rad) containing β-mercaptoethanol was added to 40 μg protein, and boiled at 95 °C for 5 min. Protein were separated by SDS–PAGE, transferred to nitrocellulose membranes (Millipore) overnight, blocked with 5% bovine serum albumin in PBS containing 0.05% Tween-20 for 1 h and incubated with primary antibody overnight at 4 °C. Primary antibodies used were: rabbit anti-KLF4, rabbit anti-SMAD2/3, rabbit anti-GAPDH (1:500, 1:500, 1:5000; Cell Signaling Technology), rabbit anti-phospho-SMAD3, rabbit anti-collagen 1, rabbit anti-CCL2, rabbit anti-FOXM1 (1:500, 1:1000, 1:1000, 1:1000; Abcam), mouse anti-SMA (1:2000, Sigma) and mouse anti-fibronectin (1:5000, BD Biosciences). After washing, membranes were incubated with horseradish peroxidase–conjugated secondary antibodies (1:2000; DAKO) for 1 h. Detection was performed with the Western Blotting Substrate Plus (Pierce) and GBOX imaging system (Syngene).

### Cell culture

Cells were cultured on dishes coated with 0.1% gelatin, and cells of passages 2–4 were used for experiments. Zs^+^ cells isolated from the lungs of *Pdgfrb*-*CreER*^*T2*^, *ROSA26R*^(*Zs*/+)^ mice were cultured in low glucose DMEM (Gibco) supplemented with 10% FBS, 1% penicillin/streptomycin (Life Technologies), 5 μg/ml gentamycin (Sigma-Aldrich) and 4 μg/ml amphotericin B (Corning^TM^). Prior to staining, Zs^+^ cells were fixed in 4% PFA and permeabilized in 0.5% Triton X-100 in PBS. Primary mouse airway SMCs (Cell Biologics) were cultured in SMC medium (Cell Biologics) supplemented with EGF and FGF, 10% FBS and 1% penicillin/streptomycin.

### siRNA-mediated knockdown of Klf4

Murine lung PDGFR-β^+^ cells or airway SMCs were serum starved for 2 h and then transfected with Lipofectamine RNAiMAX or Lipofectamine 2000 (Invitrogen) containing siRNAs (Origene or Dharmacon) targeted against Klf4 (50 nM) or Scr RNA for 6 h. Cells were washed in PBS and cultured in supplemented SMC medium for 72 h. The conditioned medium was then collected, and cells were washed in PBS. Cell lysates were subjected to western blot or RNA was isolated for qRT-PCR analysis.

### Conditioned medium experiments

Conditioned medium from Scr RNA or siKlf4-treated SMCs was centrifuged at 940 × *g* for 5 min, and the supernatant was collected. Isolated Zs^+^ cells from the lungs of *Pdgfrb*-*CreER*^*T2*^, *ROSA26R*^(*Zs*/+)^ mice were serum starved for 2 h, washed two times in PBS and incubated with the conditioned medium supernatant for 24 h prior to collection for qRT-PCR, dot blot or proliferation assay. For proliferation studies, during the last 8 h of this incubation, EdU (10 μm) from the Click-iT EdU Alexa Fluor 555 Imaging Kit (Thermo Fisher Scientific) was added to the conditioned medium supernatant. Cells were then fixed in 4% PFA overnight, permeabilized in 0.5% Triton X-100 in PBS and costained for EdU and other proteins per the manufacturer’s instructions.

### Quantitative real-time PCR analysis

Cells were lysed, and RNA was isolated per the PureLink^TM^ RNA Mini Kit (Invitrogen). The iScript cDNA Synthesis Kit (Bio-Rad) was used for reverse transcription of 200 ng RNA. The mRNA levels of selected genes were determined by qRT-PCR and normalized to Gapdh. Forward and reverse primer pairs for the assayed genes are listed in Supplementary Table 1.

### Dot blot

Dot blot apparatus was assembled with nitrocellulose membrane and filter paper, following instructions of the manufacturer (Bio-Rad). SMC conditioned medium supernatant (100 μl) was applied to each Dot blot well, and the nitrocellulose membrane was allowed to dry for 1 h. The membrane was then blocked in 5% dry milk in tris-buffered saline (TBS) containing 0.1% Tween-20, and incubated in rabbit anti-CCL2 antibody (1:1000, Abcam) overnight at 4 °C. The next day, after washing in TBS with 0.1% Tween-20, the membrane was incubated with horseradish peroxidase–conjugated anti-rabbit secondary antibody (1:2000; DAKO) for 1 h. Detection was performed with the Western Blotting Substrate Plus (Pierce) and GBOX imaging system (Syngene).

### Chromatin immunoprecipitation

Eight confluent 10-cm dishes of PDGFR-β^+^ cells or SMCs (~8 × 10^6^ cells) were treated with 1% formaldehyde for 10 min to cross-link protein to DNA and were neutralized with glycine. Cells were scraped in cold PBS and subjected to ChIP assay using the SimpleChIP Plus Enzymatic Chromatin IP Kit (Cell Signaling) with an antibody targeting KLF4 (Abcam) or Histone H3K9Ac (Cell Signaling). Coprecipitated DNA was analyzed by qPCR with primer sets (sense and antisense) targeting the promoter locus of *Foxm1* (5′-GCCGATTGGCGACGCT-3′ and 5′-CGCCGCTTTCAGTTGTTCCG-3′) or *Tgfb1* (5′-CCTTGACACTCTCATCCGCA-3′ and 5′-TGGGACTTCGTGAGAAAACAGA-3′). For ChIP assay with the anti-KLF4 antibody, the amount of coprecipitated DNA was normalized to that from ChIP using control rabbit IgG (1:500, Cell Signaling). For ChIP assay with the H3K9Ac antibody, the amount of coprecipitated DNA in PDGFR-β^+^ cells is relative to the amount of PCR product amplified from input samples reserved before immunoprecipitation and then normalized to this value in SMCs.

### Imaging

Images were acquired with Leica SP5 or SP8 confocal microscope, PerkinElmer UltraView Vox Spinning Disc confocal microscope or Nikon Eclipse 80i upright fluorescent microscope. For image processing, analysis and cell counting, Volocity software (PerkinElmer), Image J and Adobe Photoshop were used.

### Statistical analysis

Student’s *t* test, one-way ANOVA with Tukey’s multiple comparisons test were used to analyze the data (Prism 7 software). Statistical significance threshold was set at *p* ≤ 0.05. Tests assumed normal distribution and were two-sided, and all data are presented as mean ± standard deviation.

### Study approval

All mouse experiments were approved by the IACUC at Yale University and were performed in accordance with relevant ethical regulations. All procedures involving human subjects were approved by the Institutional Review Board of Yale University (IRB# 1307012431), and we complied with all relevant ethical regulations. Written informed consent was obtained from all participants and/or a family member prior to inclusion in the study.

### Reporting summary

Further information on research design is available in the Nature Research Reporting Summary linked to this article.

## Supplementary information

Supplementary Information

Description of Additional Supplementary Files

Supplementary Data 1

Supplementary Data 2

Supplementary Data 3

Reporting Summary

## Data Availability

Data are available in the main figures, Supplementary Figures and Supplementary Data. The source data underlying Figs. 1c, d, f, h, i, 2b, d, e, g, i, 3c–e, g, i, 4a–h, 5c–e, g, i, 6a–f, h, j–l and 7d–l and Supplementary Figs. 5b, 8, 9b, c, 11a, c, 13a, c, 14a, b and 15b are provided as a Source Data file. The TRANSFAC database is publicly available (https://portal.genexplain.com/cgi-bin/portal/login.cgi). The bulk and scRNA-seq data generated in this study have been deposited in the Gene Expression Omnibus database under accession code GSE184672. Source data are provided with this paper.

## Source data

Source Data
