# Supplementary material for: Distinct roles of KLF4 in mesenchymal cell subtypes during lung fibrogenesis
Source: Nat Commun. 2021 Dec 10;12:7179. doi: 10.1038/s41467-021-27499-8 (PMC8664937; doi:10.1038/s41467-021-27499-8)
Supplement: Supplementary file 1 — Supplementary Information [file 41467_2021_27499_MOESM1_ESM.pdf]

SUPPLEMENTARY INFORMATION

Distinct roles of KLF4 in mesenchymal cell subtypes during lung fibrogenesis

Rachana R. Chandran, Yi Xie, Eunate Gallardo-Vara, Taylor Adams, Rolando Garcia-Milian, Inamul Kabir, Abdul Q. Sheikh, Naftali Kaminski, Kathleen A. Martin, Erica L. Herzog and Daniel M. Greif

List of Supplementary Items:

- Supplementary Figures with Legends 1-15
- Supplementary Methods
- Supplementary Table 1
- Supplementary Data 1-3

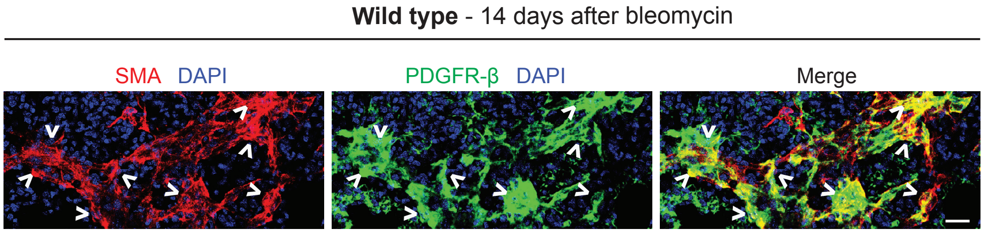

Supplementary Figure 1. The vast majority of myofibroblasts in bleomycin-induced lung injury express PDGFR-β. Wild type mice were given a single orotracheal dose of bleomycin and lungs were harvested 14 days later. Lung cryosections were stained with SMA, PDGFR-β and nuclei (DAPI). Open arrowheads indicate PDGFR-β+ myofibroblasts. n=3 mice. Scale bar, 25 μm.

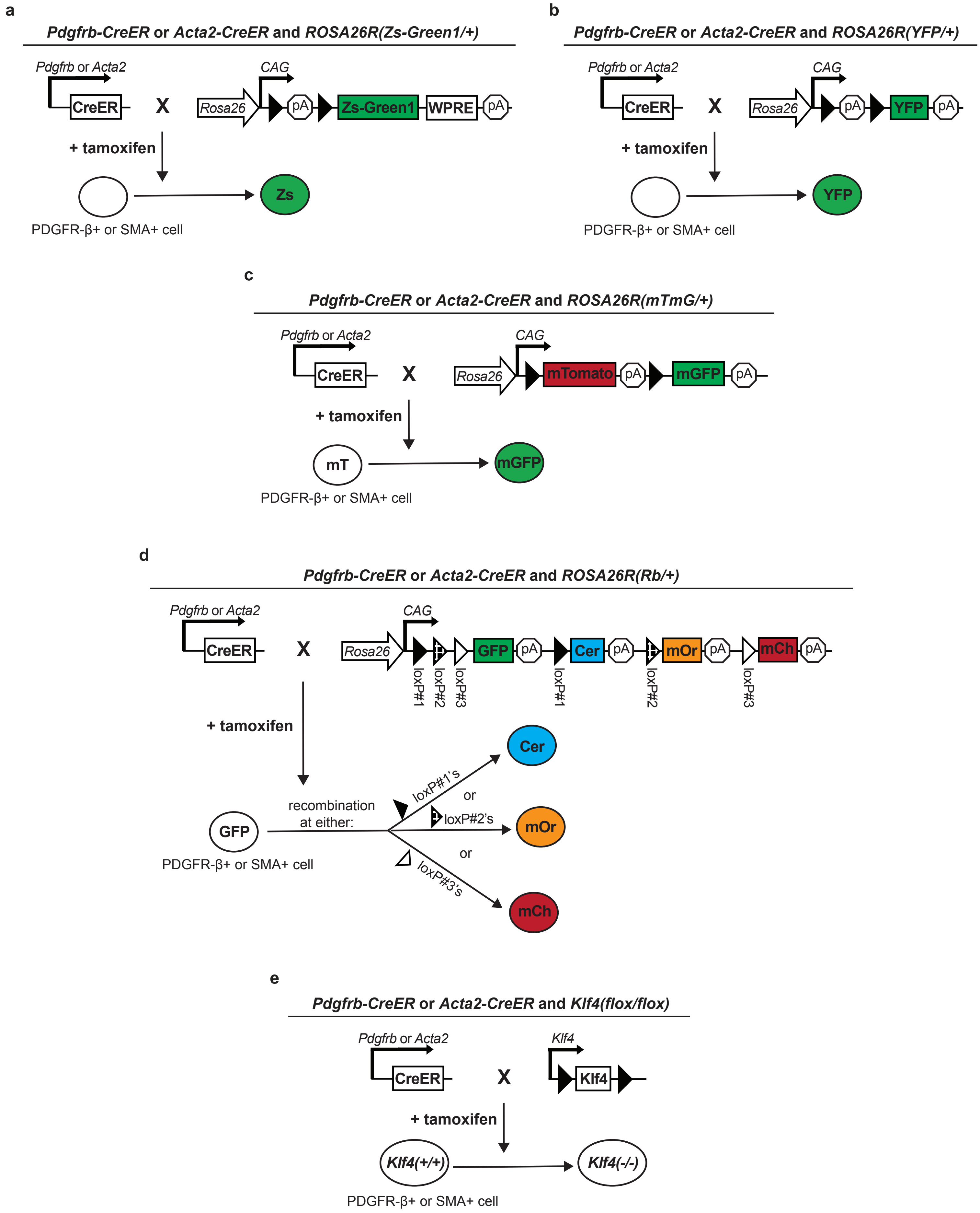
a
Pdgfrb-CreER or Acta2-CreER and ROSA26R(Zs-Green1/+)
Pdgfrb or Acta2
CreER
X
CAG
Rosa26
pA
Zs-Green1
WPRE
pA
+ tamoxifen
Zs
PDGFR-β+ or SMA+ cell
b
Pdgfrb-CreER or Acta2-CreER and ROSA26R(YFP/+)
Pdgfrb or Acta2
CreER
X
CAG
Rosa26
pA
YFP
pA
+ tamoxifen
YFP
PDGFR-β+ or SMA+ cell
c
Pdgfrb-CreER or Acta2-CreER and ROSA26R(mTmG/+)
Pdgfrb or Acta2
CreER
X
CAG
Rosa26
mTomato
pA
mGFP
pA
+ tamoxifen
mT
mGFP
PDGFR-β+ or SMA+ cell
d
Pdgfrb-CreER or Acta2-CreER and ROSA26R(Rb/+)
Pdgfrb or Acta2
CreER
X
CAG
Rosa26
GFP
pA
Cer
pA
mOr
pA
mCh
pA
loxP#1
loxP#2
loxP#3
loxP#1
loxP#2
loxP#3
+ tamoxifen
recombination at either:
GFP
PDGFR-β+ or SMA+ cell
loxP#1's
or
loxP#2's
or
loxP#3's
Cer
mOr
mCh
e
Pdgfrb-CreER or Acta2-CreER and Klf4(flox/flox)
Pdgfrb or Acta2
CreER
X
Klf4
Klf4
+ tamoxifen
Klf4(+/+)
Klf4(-/-)
PDGFR-β+ or SMA+ cell

Supplementary Figure 2. Schematic of genetic strategies for cell marking or *Klf4* deletion in mice. a-d, *Pdgfrb-CreERT2* or *Acta2-CreERT2* mice also carrying one allele of a Cre reporter - Zs-Green1 (Zs), YFP, mTomato mGFP (mTmG) or Rainbow (Rb) - knocked into the *ROSA26* locus were used for inducible cell marking in investigations herein. The CAG promoter consists of a cytomegalovirus enhancer, chicken *beta actin* gene promoter and splice acceptor of the rabbit *beta globin* gene. The woodchuck hepatitis virus posttranscriptional regulatory element (WPRE) enhances mRNA transcript stability. Cer, Cerulean; mOr, membrane Orange; mCh, membrane Cherry. **e,** *Pdgfrb-CreERT2* or *Acta2-CreERT2* mice also carrying *Klf4(flox/flox)* were used for inducible *Klf4* deletion in PDGFR-β+ or SMA+ cells.

Pdgfrb-CreER, ROSA26R(Zs/+) - single cell analysis

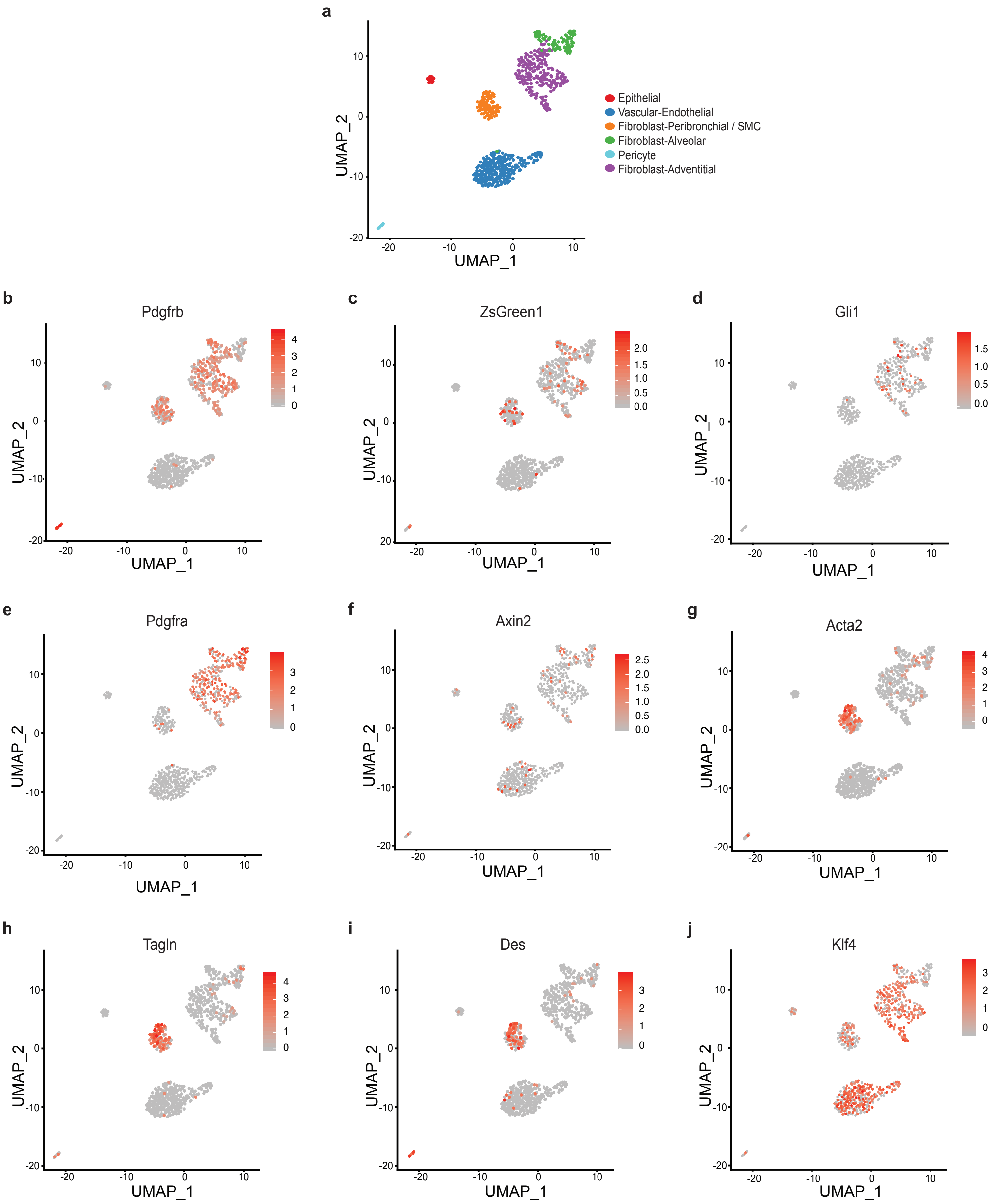

Supplementary Figure 3. scRNA-seq analysis of stromal cells in the *Pdgfrb-CreERT2, ROSA26R(Zs/+)* murine lung under basal conditions. *Pdgfrb-CreERT2, ROSA26R(Zs/+)* mice were induced with tamoxifen and rested. Lung cells were subjected to scRNA library construction (10X Genomics), sequencing and then annotating and clustering. Uniform manifold approximation and projection (UMAP) of 720 non-immune lung cells is shown. **a,** Cells are colored by their categorized cell-type identity. **b-g,** The same UMAP, colored by normalized expression levels of Pdgfrb, ZsGreen1 (Zs) reporter, Gli1, Pdgfra, Axin2, Acta2, Tagln, Des and Klf4 as indicated. Unique molecular identifiers are scaled to ten thousand transcripts per cell and then natural log transformed with a pseudocount of one.

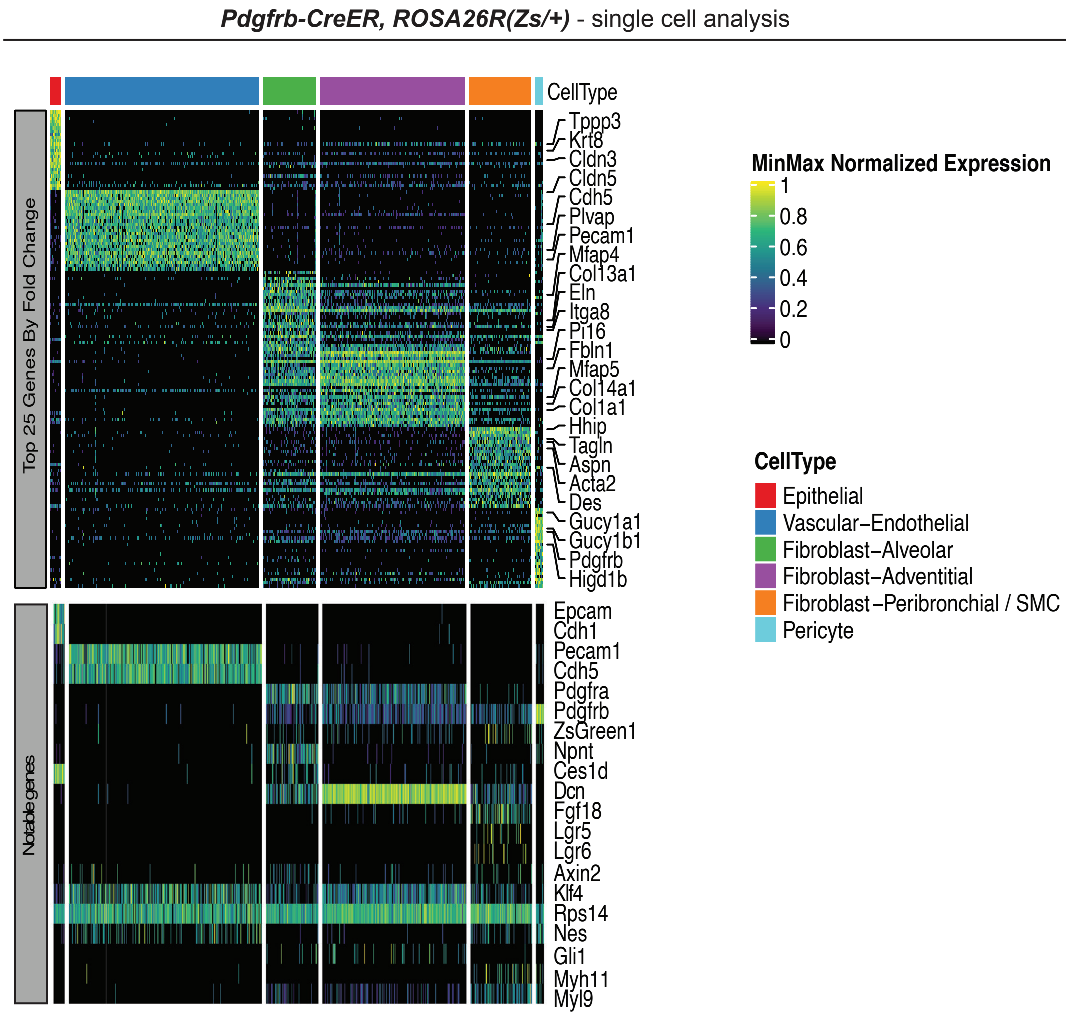

Supplementary Figure 4. Heatmap representation of gene expression signatures across cells for scRNA-seq analysis described in Supplementary Figure 3. Each column represents one cell with columns grouped by their assigned cell-type identity. Each row represents expression for one gene. The upper section of the heatmap represents the top 25 marker genes for each cell-type ranked by fold change. The lower section represents expression of canonical lineage markers, ZsGreen1 reporter and other notable genes. Log transformed, scaled expression values for each gene are unity normalized between values of 0 and 1 across all cells (columns).

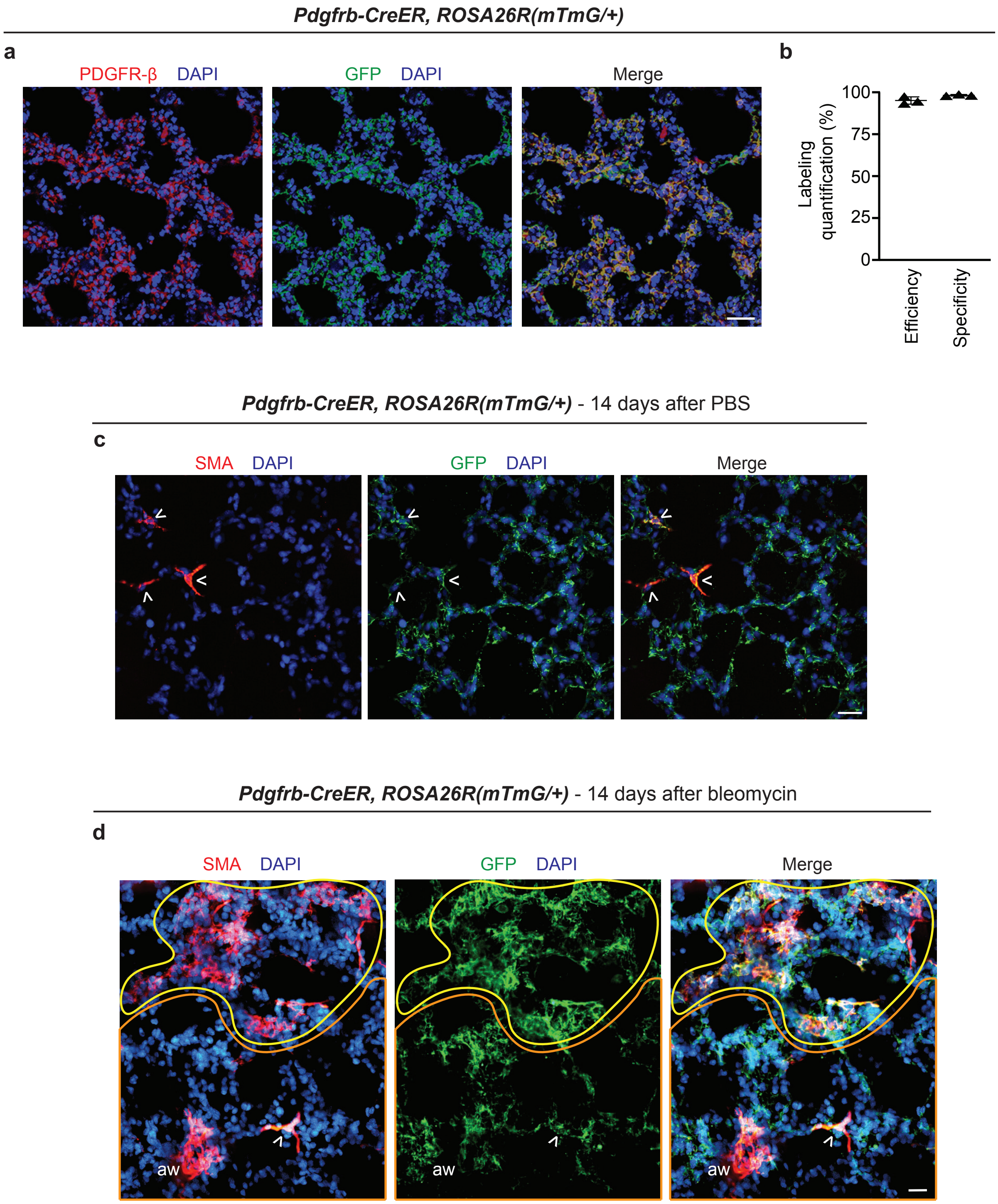

Supplementary Figure 5. Cell marking with *Pdgfrb-CreERT2.* *Pdgrb-CreERT2, ROSA26R(mTmG/+)* mice were induced with tamoxifen and rested. In **a,** mice were then euthanized. In **c, d,** mice were given a single orotracheal dose of PBS (**c**) or bleomycin (**d**) and euthanized fourteen days later. Lungs were stained for GFP (fate marker), nuclei (DAPI) and either PDGFR-β or SMA. In **d,** fibrotic and non-fibrotic regions are indicated by yellow and orange demarcations, respectively. In **b,** stains represented by **a** were used to determine the labeling efficiency (% of PDGFR-β+ cells that are GFP+) and specificity (% of GFP+ cells that are PDGFR-β+). n=3 mice, 3 sections per mouse, an average of 263 cells were analyzed per section. Arrowheads indicate myofibroblasts. aw, airway. Data are averages +/- SD. Source data are provided as a Source Data file. Scale bars, 25 μm.

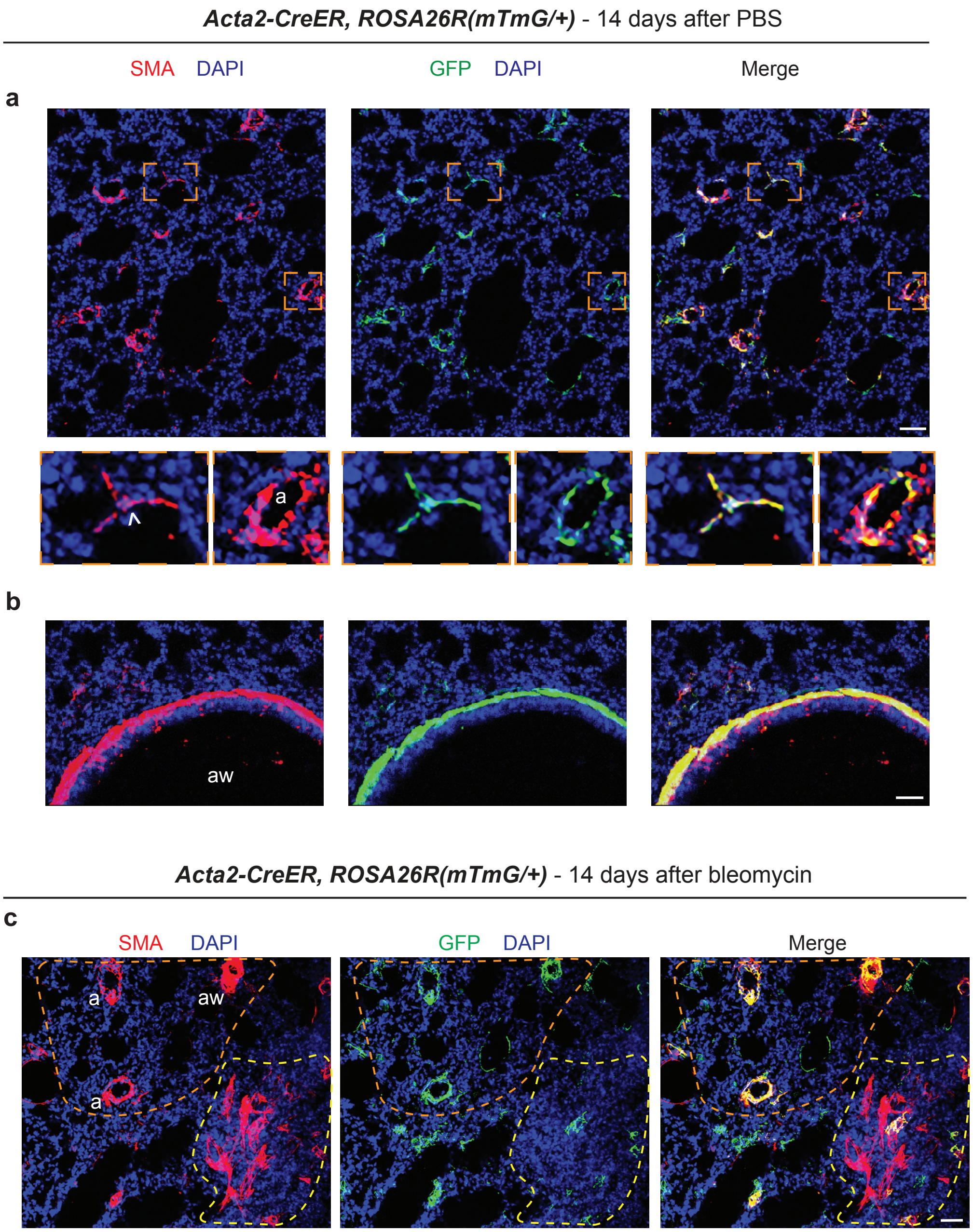

Supplementary Figure 6. Cell marking with *Acta2-CreERT2*. *Acta2-CreERT2, ROSA26R(mTmG/+)* mice were induced with tamoxifen, rested, injected with a single orotracheal dose of PBS (**a, b**) or bleomycin (**c**) and euthanized fourteen days later. Lungs were stained for SMA, GFP (fate marker) and nuclei (DAPI). In **a,** boxed regions are shown as close-ups below. In **c,** fibrotic and non-fibrotic regions are indicated by yellow and orange demarcations, respectively. n=3 mice. Scale bars, 50 µm.

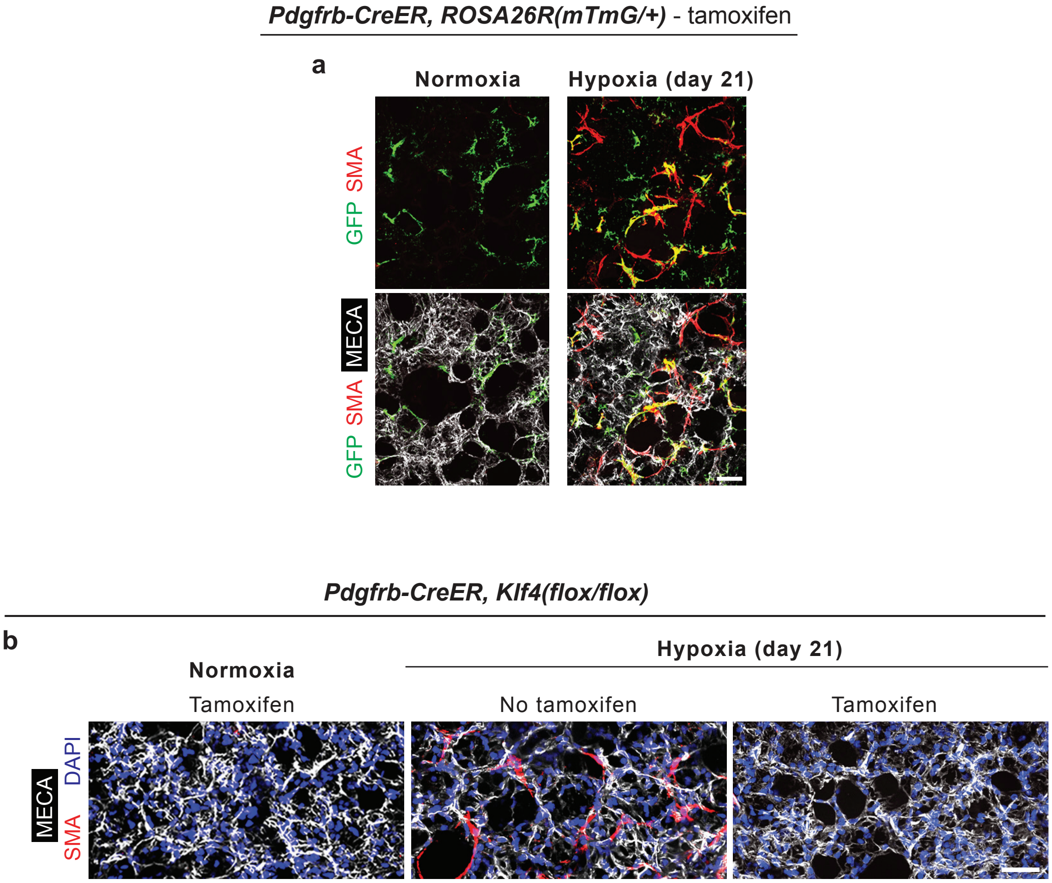

Supplementary Figure 7. With hypoxia, PDGFR-β+ cells give rise to many lung myofibroblasts and *Klf4* deletion attenuates myofibroblast accumulation. *Pdgfrb-CreERT2* mice were or were not induced with tamoxifen as indicated and then rested and exposed to normoxia or hypoxia (FiO2 10%) for 21 days. Lung vibratome sections were analyzed. In **a,** mice also carrying *ROSA26R(mTmG/+)* were induced with tamoxifen, and sections were stained for SMA, GFP (fate marker) and MECA-32 (ECs). Per treatment group, n=2 mice, 8 sections per mouse were analyzed. In **b,** sections from mice also carrying *Klf4(flox/flox)* were stained for SMA, MECA-32 and nuclei (DAPI). For each experimental group, n=3 mice and at least 10 sections per mouse were stained. Scale bars, 25 μm.

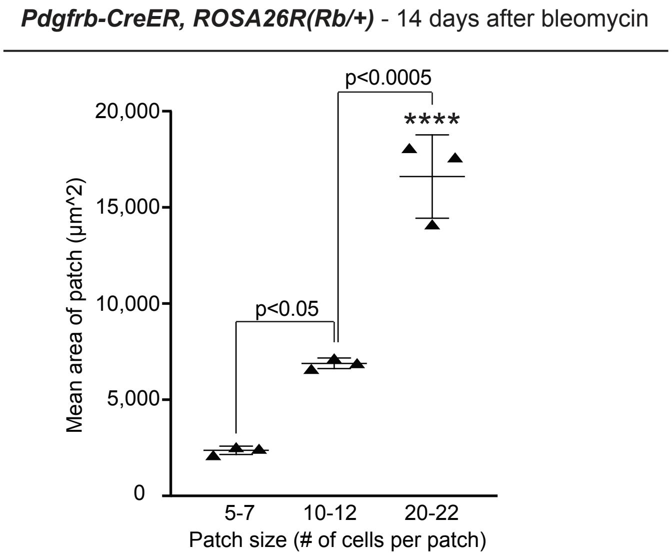

Supplementary Figure 8. Following bleomycin treatment, patches of clones with more cells cover a larger area than clones with fewer cells. *Pdgfrb-CreERT2, ROSA26R(Rb/+)* were labelled with tamoxifen, rested, subjected to a single orotracheal bleomycin dose and euthanized 14 days later. Lung cryosections were stained with DAPI and directly imaged for Rb colors (mCherry, mOrange, Cerulean). Among clones consisting of 5-7, 10-12 or 20-22 cells, clones were chosen randomly and the clonal patch area was measured. n=3 mice, 5 patches per each clone cell size per mouse. One-way ANOVA with Tukey's multiple comparisons test was used. Patch size 5-7 vs. 10-12 (p=0.011), 10-12 vs. 20-22 (p=0.0002), *** vs. 5-7 (p<0.0001). Data are averages +/- SD. Source data are provided as a Source Data file.

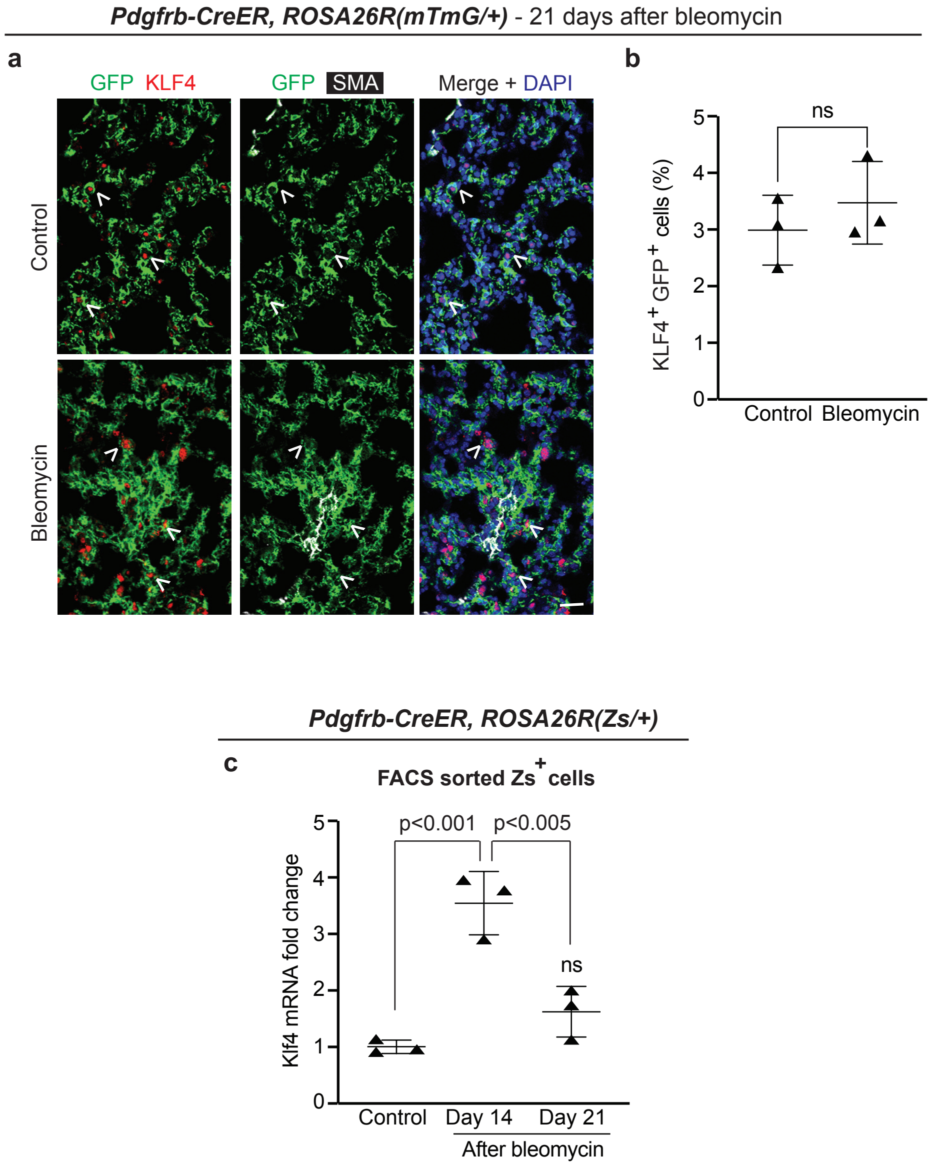

Supplementary Figure 9. KLF4 expression in the lineage of PDGFR-β+ cells in the lung does not differ significantly between untreated mice or day 21 after bleomycin treatment. *Pdgfrb-CreERT2* mice carrying *ROSA26R(mTmG/+) or ROSA26R(Zs/+)* as indicated were induced with tamoxifen, rested and then not treated (control) or treated with an orotracheal bleomycin dose. **a,** Twenty-one days later, mice were euthanized, and lung cryosections were stained for KLF4, GFP (fate marker), SMA and nuclei (DAPI). Arrowheads indicate KLF4+GFP+ cells. Scale bar, 25 µm. **b,** Quantification of the percentage of GFP+ cells that express KLF4 are shown. n=3 mice, 5 sections per mouse, and on average 393 GFP+ cells per section were scored. Two-tailed Student's t-test was used, ns (not significant). **c,** Fourteen or twenty-one days following bleomycin or no treatment, Zs+ cells were isolated by FACS and subjected to qRT-PCR. Levels of Klf4 transcript are relative to Gapdh and normalized to no treatment. For each treatment group and time point, n=3 mice in triplicate. One-way ANOVA with Tukey's multiple comparisons was used. Control vs. day 14 (p=0.0008), day 14 vs. day 21 (p=0.0033), ns vs. control. Data are averages +/- SD. Source data are provided as a Source Data file.

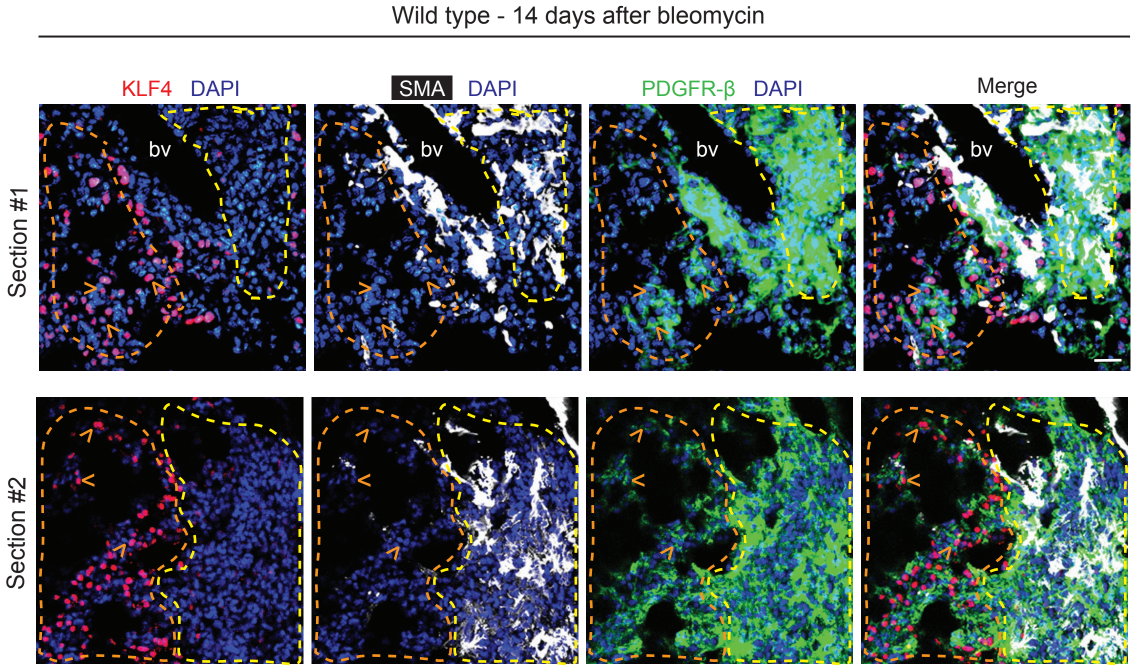

Supplementary Figure 10. KLF4 is down-regulated in SMA+ pathological myofibroblasts. Fourteen days after a single orotracheal dose of bleomycin, wild type mice were euthanized and lung cryosections were stained for KLF4, SMA, PDGFR-β and nuclei (DAPI). Sections #1 and #2 are representative images from different mice; n=6 mice. Orange and yellow lines enclose areas with minimal SMA+ myofibroblasts and highly fibrotic areas with SMA+ myofibroblasts, respectively. Arrowheads indicate PDGFR-β+KLF4+ cells in low fibrotic areas. Scale bar, 25 µm.

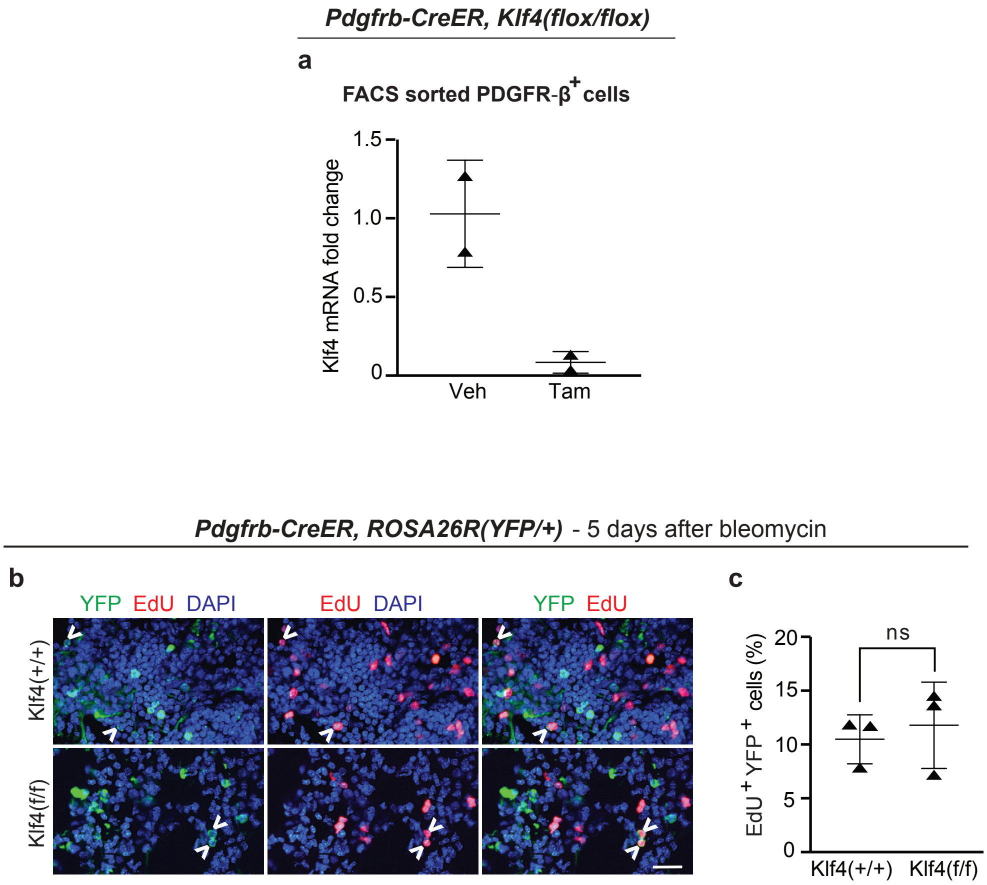

Supplementary Figure 11. *Klf4* deletion in lung PDGFR-β+ cells is efficient but does not change bleomycin-induced proliferation of PDGFR-β-derived lung cells at day 5. a, *Pdgfrb-CreERT2, Klf4(flox/flox)* mice were treated with vehicle (Veh; corn oil) or tamoxifen (Tam) and then rested. Lung PDGFR-β+ cells were isolated by FACS, and Klf4 mRNA levels were assayed by qRT-PCR. n=2 mice for each treatment with qRT-PCR done in triplicate. **b, c,** *Pdgfrb-CreERT2, ROSA26R(YFP/+)* mice also carrying *Klf4(flox/flox)* or wild type for *Klf4* were induced with tamoxifen, rested, treated with a single orotracheal dose of bleomycin and then euthanized five days later. EdU was administrated 12 hours before euthanasia. In **b,** lung cryosections were stained for YFP (fate marker), EdU and nuclei (DAPI) with arrowheads indicating proliferating YFP+ cells. Scale bar, 25 μm. In **c,** percent of YFP+ cells that are EdU+ is shown. For each genotype, n=3 mice, 3 sections per mouse and an average of 48 YFP+ cells were analyzed per section. Two-tailed Student’s t-test; ns, not significant. Data are averages +/- SD. Source data are provided as a Source Data file.

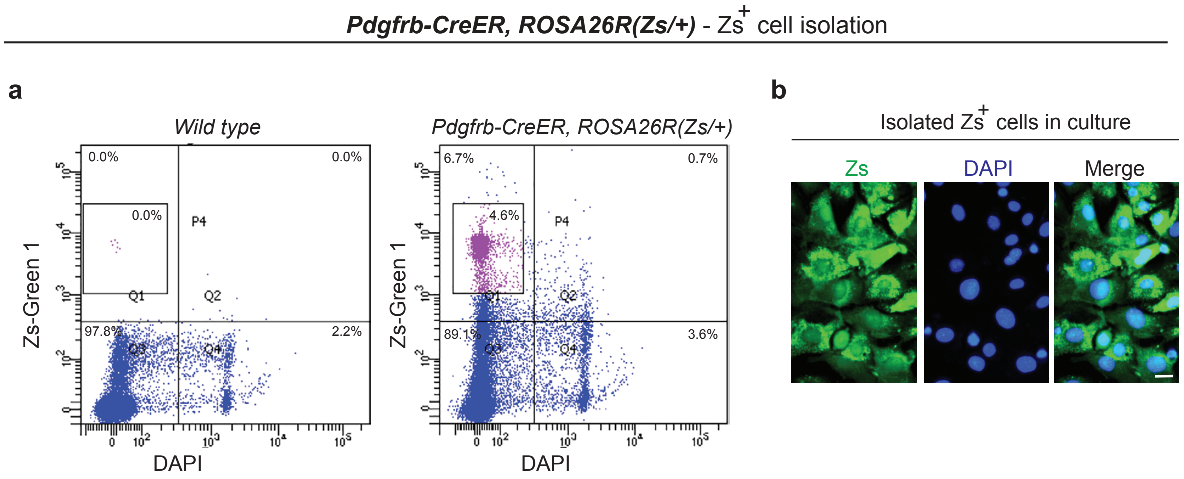

Supplementary Figure 12. Isolation of Zs+ cells from the lungs of *Pdgfrb-CreERT2, ROSA26R(Zs/+)* mice. Lungs were harvested from wild type and tamoxifen-injected *Pdgfrb-CreERT2, ROSA26R(Zs/+)* mice, and a single cell suspension was generated. Cells were stained with DAPI, and Zs+DAPI- cells were isolated by FACS. **a,** FACS plots of wild type (left panel) and *Pdgfrb-CreERT2, ROSA26R(Zs/+)* (right panel) mice. Lung cells from wild type mice were used to control for auto-fluorescence and set gating for Zs+ cell isolation. Box within quadrant #1 (Q1) indicates the isolated Zs+DAPI- cells. n=5. **b,** Isolated Zs+ cells were cultured for two weeks and then stained for nuclei (DAPI) and directly imaged for Zs. n=5. Scale bar, 25 µm.

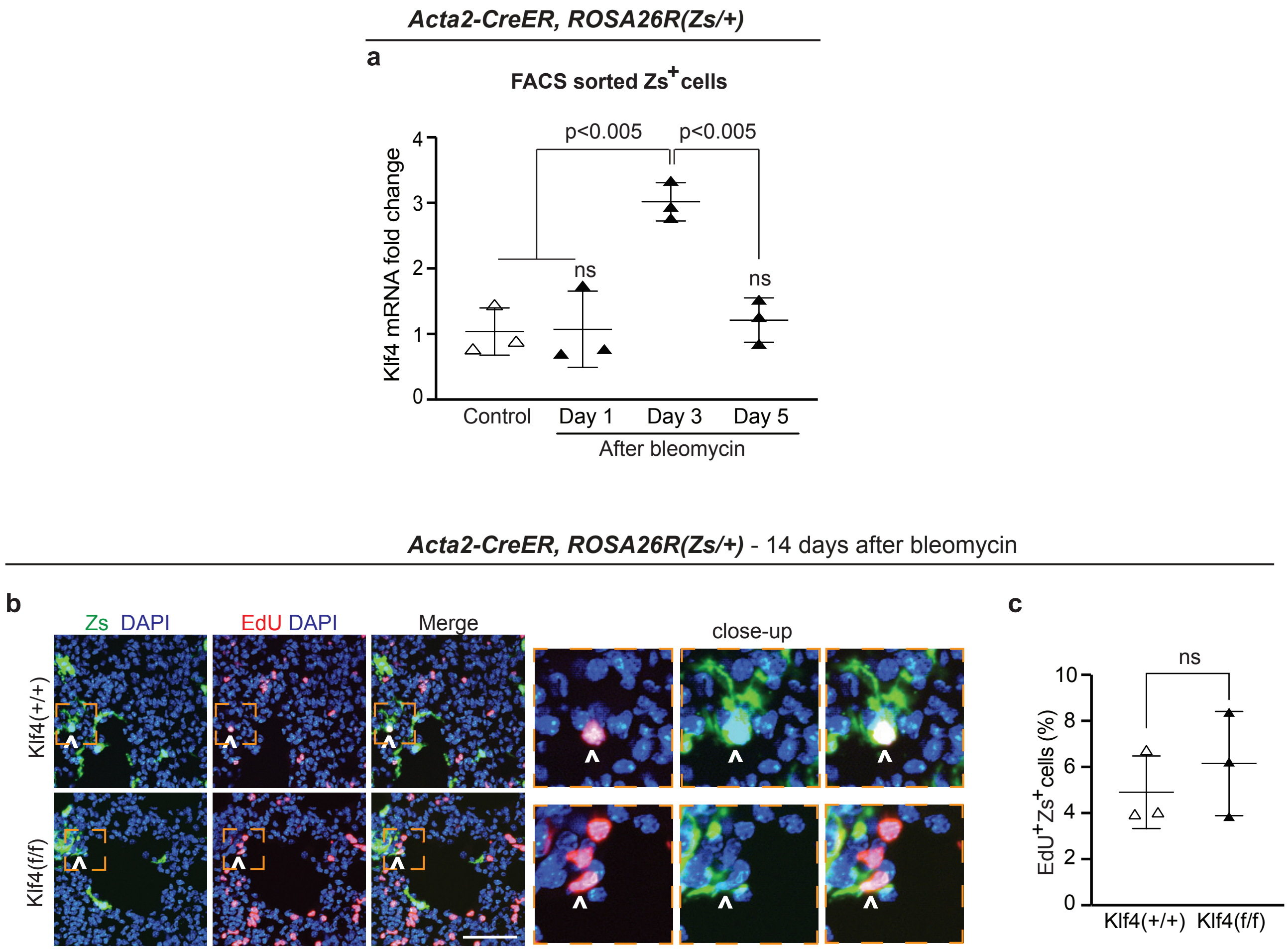

Supplementary Figure 13. With bleomycin administration, KLF4 is dynamically upregulated in SMA+ lung cells but Klf4 deletion in SMA+ cells does not alter proliferation of this lineage. *Acta2-CreERT2, ROSA26R(Zs/+)* mice were induced with tamoxifen and rested. **a,** Mice were or were not treated with a single orotracheal dose of bleomycin, and at 1, 3 and 5 days later, Zs+ cells were isolated by FACS and subjected to qRT-PCR for Klf4 and Gapdh. Transcript levels of Klf4 relative to that of Gapdh for each time point and normalized to control (no treatment) are shown. For each time point, n=3 mice in triplicate. One-way ANOVA with Tukey's multiple comparisons test was used. Control vs. day 3 (p=0.0018), day 3 vs. day 5 (p=0.0033). ns, not significant vs. control. **b, c,** Mice were also carrying *Klf4(flox/flox)* or were wild type for *Klf4*. EdU was administrated 12 hours before mice were euthanized on day 14 after bleomycin, and lung sections were stained for EdU and nuclei (DAPI) and directly imaged for Zs. In **b,** boxed areas are shown as close-ups on the right, and arrowheads indicate EdU+Zs+ cells. Scale bar, 25 μm. In **c,** the percentage of Zs+ cells that are EdU+ is shown for *Klf4* wild type and floxed groups. For each genotype, n=3 mice, 3 sections per mouse and an average of 67 Zs+ cells per section were analyzed. Two-tailed Student's t-test was used. Data are averages +/- SD. Source data are provided as a Source Data file.

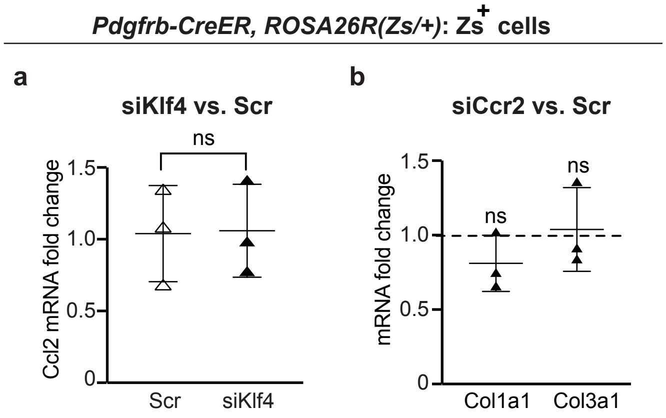

Supplementary Figure 14. siRNA mediated knockdown of lung Zs+ cells isolated from *Pdgfrb-CreERT2, ROSA26R(Zs/+)* mice. Zs+ cells were isolated by FACS from the lungs of tamoxifen-induced *Pdgfrb-CreERT2, ROSA26R(Zs/+)* mice. Cells were then subjected to scrambled (Scr) RNA or siRNA-mediated knockdown of Klf4 (**a**) or Ccr2 (**b**). Transcript levels of Ccl2 (**a**) or Col1a1 and Col3a1 (**b**) were determined by qRT-PCR relative to Gapdh and normalized to Scr. n=3 in triplicate. Two-tailed Student's t-test was used. ns, not significant. Data are averages +/- SD. Source data are provided as a Source Data file.

a

Bulk RNA-seq

	SMC		PDGFR-beta+	
**Gene Symbol**	**Log2 FC**	**FDR (p-adj)**	**Log2 FC**	**FDR (p-adj)**
Klf4	-2.20	6.35x10^(-38)	-3.56	0
Pdk1	0.74	2.56x10^(-18)	-0.52	1.29x10^(-4)

b

qRT-PCR

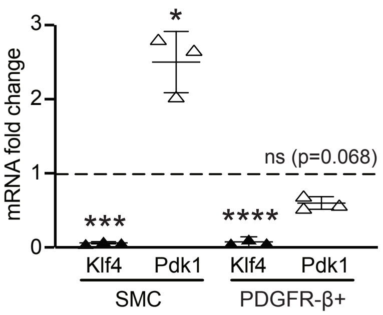

Supplementary Figure 15. Klf4 silencing increases Pyruvate dehydrogenase kinase (Pdk) 1 transcript levels in SMCs but not PDGFR-β+ cells. Murine lung SMCs or Zs+ cells isolated from the lungs of tamoxifen-induced *Pdgfrb-CreERT2, ROSA26R(Zs/+)* mice were subjected to Scr RNA or siKlf4 treatment. Transcript levels of Klf4 and Pdk1 were determined by bulk RNA-seq (**a**) or qRT-PCR (**b**). In **b,** transcript levels were relative to that of Gapdh. In **a, b,** results of siKlf4 treatment were normalized to results of Scr treatment. n=3 per condition, each n in **b** was done in triplicate. FC, fold change. FDR, false discovery rate. For **a,** DESeq2 was used which employs two-sided Wald test to determine the p-value and Benjamini-Hochberg FDR for p-adjusted value. For **b,** two-tailed Student's t-test was used; *, p=0.011; ***, p=0.0004; ****, <0.0001; ns (not significant, p=0.068) vs. Scr. Data are averages +/- SD. Source data are provided as a Source Data file.

Supplementary Methods

Sample preparation for single-cell RNA sequencing

Finely minced lung tissue was incubated at 37°C for 40 min in an enzyme mixture from the Lung Dissociation Kit (Miltenyi Biotec) as per the manufacturer's instructions. At 20 min intervals during this incubation, tissue was subjected to the gentle lung dissociation protocol using a gentleMACS dissociator (Miltenyi Biotec). Enzymatic activity was then inhibited by adding 10% FBS in PBS. Cell suspensions from three mice were pooled and passed through a 70 μm cell strainer and centrifuged at 700 ×g for 10 min at 4°C. Cells were stained with DAPI (nuclear stain) and Zs^+ $DAPI^-$ cells and Zs^- $DAPI^-$ cells were isolated by FACS. Isolated Zs^+ and Zs^- cells were hash-tagged separately following manufacturer's instructions (Biolegned), stained with Trypan blue and then counted on a Countess Automated Cell Counter (Thermo Fisher Scientific) to assess single cell suspension and viability. The cells were then pooled together in PBS with 0.04% bovine serum albumin and then subjected to library preparation and DropSeq.

Construction of 10X Genomics single cell 3' RNA-seq libraries and sequencing:

a) Gel Beads-In-Emulsion generation and barcoding

Single cell suspension in RT Master Mix (75 μl) was loaded on the Single Cell A Chip and partitioned with a pool of ~750,000 barcoded gel beads to form nanoliter-scale Gel Beads-In-Emulsions (GEMs). Each gel bead has primers containing: (i) an Illumina R1 sequence (read 1 sequencing primer), (ii) a 16 nt 10x Barcode, (iii) a 10 nt Unique Molecular Identifier (UMI), and (iv) a poly-dT primer sequence. Upon dissolution of the Gel Beads in a GEM, the primers

were released and mixed and incubated with cell lysate and RT Master Mix, producing barcoded, full-length cDNA from poly-adenylated mRNA.

b) Post GEM reverse transcription cleanup, cDNA amplification and library construction

Silane magnetic beads were used to isolate cDNA from leftover biochemical reagents and primers in the post GEM reaction mixture. Full-length, barcoded cDNA was then amplified by PCR to generate sufficient DNA for library construction. Enzymatic fragmentation and size selection were used to optimize cDNA amplicon size prior to library construction. R1 (read 1 primer sequence) were added during GEM incubation. P5, P7, a sample index, and R2 (read 2 primer sequence) were added during library construction via end repair, A-tailing, adaptor ligation and PCR. The final libraries contain the P5 and P7 primers used in Illumina bridge amplification.

c) Sequencing libraries

The single cell 3' library comprised standard Illumina paired-end constructs which begin and end with P5 and P7. The single cell 3' 16 bp 10x Barcode and 10 bp UMI were encoded in Read 1, and Read 2 was used for sequencing the cDNA fragment. Sequencing the single cell 3' library produced standard Illumina Binary Base Call data which includes the paired-end Read 1 and Read 2 and the sample index in the i7 index read.

Processing scRNA-seq data

scRNA-seq data was processed with Cell Ranger v3.1.0. 62.7% of reads were confidently aligned to a modified version of the mouse transcriptome mm10 that includes the sequence for

the gene ZsGreen1. The top cell barcodes selected by Cell Ranger were then utilized for downstream analysis.

Cell barcode clustering and annotation

All analyses were performed in R version 3.6.1. Graph embedding and clustering were performed using the R package Seurat v3.1.0. UMI counts were scaled to 10,000 UMIs per cell, then natural log transformed with a pseudo-count of one (log((TPM/100) + 1)). Feature selection, principal component analysis, neighbor embedding and Louvain modularity clustering were recursively performed on the data. This analysis was conducted to identify discrete clusters of cell populations with consistent gene signatures in each cluster and distinctness between clusters. Clusters were then annotated as either a multiplet population or known cell type by curation of transcriptional marker genes. Mesenchymal cell-type nomenclature was assigned to clusters in concordance with findings previously reported by Tsukui et al (ref [#37] in main text). Multiplets were identified as cell populations whose transcriptomic profile resembled a combination of two or more cell populations found in the data. Markers previously shown to be enriched in peribronchial mesenchymal cells (Hhip, Aspn) or in airway/vascular SMCs (Myh11, Acat2) (ref [#37]) were observed in our scRNA-seq data to not be distinctly expressed but instead co-expressed among a single population of cells that we labeled as “fibroblast-peribronchial / SMC”. These cells are unlikely to be multiplets because singlets of either population were not identified, and their absolute RNA content was similar to ordinary cells. This cell population may be representative of the lung “myofibroblast” cluster reported by Xie et al. that similarly expresses markers of SMCs (Acta2, Myh11, Tgln) as well as Hhip, Aspn (ref [#38]) and the “peribronchial fibroblast” cluster reported by Tsukui et al. (ref [#37]). Of note, similar to our results, Xie et al. did

not report a distinct SMC cluster in their scRNA-seq analysis of the lung (ref [#38]). Potentially, in contrast to Tsukui et al. (ref [#37]), we did not detect a distinct SMC cluster because of: i) cell dissociation bias; ii) effects of tamoxifen; and/or iii) cells spending a longer duration in our study in suspension between tissue digestion and droplet-encapsulation for scRNA-seq.

Following cell annotation, immune cells and multiplets were discarded; the remaining clusters of mesenchymal, endothelial and epithelial cells were then used to generate a Uniform Manifold Approximation and Projection (UMAP) embedding to visualize expression of specific transcripts. Cell hash-tagging was not included in the analysis due to poor correlation between tagging and ZsGreen1 expression.

UMAP embedding

The top 2,500 variable genes were selected with the Seurat FindVariableFeatures implementation and then scaled and used to generate principal components with the ScaleData and RunPCA implementations respectively. The final UMAP was generated by using the top 21 principal components with the Seurat RunUMAP implementation, with a neighborhood size of 10, a minimum distance parameter of 0.8 and 5,000 epochs with a learning rate of 0.5.

Marker heatmap

R package ComplexHeatmap v3.2.3.z was used to generate a heatmap. The genes in the upper part of the heatmap are the top 25 significantly upregulated genes for each cell type versus all other cells using a Wilcoxon rank-sum test on the log((TPM/100) + 1) expression data (Bonferroni adjusted p-value < 0.01; genes tested had positive natural log fold change > 0.6); ranked by average fold change.

Bulk RNA sequencing:

a) RNA isolation and quality control

Total RNA from siKlf4 or Scr RNA treated PDGFR-β^{+} cells or SMCs were obtained using PureLink™ RNA Minikit (Invitrogen). RNA quality was determined by measuring A260/A280 and A260/A230 ratios by nanodrop. RNA integrity was determined by running an Agilent Bioanalyzer gel to measure the ratio of ribosomal peaks. Samples used for library preparation had RIN values greater than 8.

b) Bulk RNA-seq library preparation

mRNA was purified from ~200 ng of total RNA with oligo-dT beads and sheared by incubation at 94°C in the presence of Mg^{2+} with the KAPA mRNA HyperPrep kit (Roche). Following first-strand synthesis with random primers, second strand synthesis and A-tailing were performed with dUTP to generate strand-specific sequencing libraries. Adapter ligation with 3' dTMP overhangs were ligated to library insert fragments. Library amplification of fragments carrying the appropriate adapter sequences at both ends was undertaken. Strands marked with dUTP were not amplified. Indexed libraries were quantified by qRT-PCR using a commercially available kit (Roche, KAPA Biosystems) and insert size distribution was determined with the Agilent Bioanalyzer. Samples with a yield of ≥ 0.5 ng/µl and a size distribution of 150-300 bp were used for sequencing.

c) Flow cell preparation, sequencing and data analysis

Samples at a concentration of 1.2 nM were loaded onto an Illumina NovaSeq6000 flow

cell to yield 25 million passing filter clusters per sample. Samples were sequenced using 100 bp paired-end sequencing per Illumina protocols. Reads were trimmed to remove low quality base calls. HISAT2 was used to align trimmed reads to the reference genome mm10 with GENCODE annotation for mouse. Gene counts and transcript abundance were estimated using ballgown/stringTie and differential gene expression analysis was performed using R-based DESeq2. The log2 fold change was calculated by dividing knockdown values by Scr values and significance is determined from 3 independent RNA-seq experiments.

Bioinformatic analysis of Ccl2 binding TFs and cross reference to bulk RNA-seq data

The sequence spanning from 5 kb upstream of the mouse *Ccl2* transcription start site to 100 bp downstream was retrieved from the UCSC Genome Browser (https://genome.ucsc.edu/ UCSC Genomics Institute, CA). This sequence was used for a TF analysis using TRANSFAC (geneXplain GmbH, Germany). TRANSFAC MATRIX TABLE, Release 2020.2 was used as matrix library, profile was set to vertebrate_non_redundant_minFP.prf, only high-quality matrices and cut-off set to minimize false positives. The resulting TFs were compared to those differentially expressed genes (DEGs) in the bulk RNA-seq data, for SMCs and PDGFR-β^{+} cells with Klf4 knockdown, having log2 fold-change $\geq \pm 1.0$ and adjusted p value < 0.05 (Benjamini-Hochberg false discovery rate) using Ingenuity Pathway Analysis (Version 52912811, Ingenuity Systems, QIAGEN). Overlapping TFs between TRANSFAC and the most upregulated genes in SMCs were assessed for subsequent analysis.

Gene	Primers
Acta2	Forward: TGCTGACAGAGGCACCACTGAA<br>Reverse: CAGTTGTACGTCCAGAGGCATAG
Col1a1	Forward: ACGGCTGCACGAGTCACAC<br>Reverse: GGCAGGCGGGAGGTCTT
Col3a1	Forward: GTTCTAGAGGATGGCTGTACTAAACACA<br>Reverse: TTGCCTTGCGTGTTTGATATTC
Ccl2	Forward: GCTCAGCCAGATGCAGTTAA<br>Reverse: TCTTGAGCTTGGTGACAAAAACT
Ccr2	Forward: GCTGTGTTTGCCTCTCTACCAG<br>Reverse: CAAGTAGAGGCAGGATCAGGCT
Fn1	Forward: CCCTATCTCTGATACCGTTGTCC<br>Reverse: TGCCGCAACTACTGTGATTCGG
Foxm1	Forward: GTCTCCTTCTGGACCATTCACC<br>Reverse: GCTCAGGATTGGGTCGTTTCTG
Gapdh	Forward: CATCACTGCCACCCAGAAGACTG<br>Reverse: ATGCCAGTGAGCTTCCCGTTCAG
IL1b	Forward: TGGACCTTCCAGGATGAGGACA<br>Reverse: GTTCATCTCGGAGCCTGTAGTG
IL5	Forward: GATGAGGCTTCCTGTCCCTACT<br>Reverse: TGACAGGTTTTGGAATAGCATTTCC
IL6	Forward: TACCACTTCACAAGTCGGAGGC<br>Reverse: CTGCAAGTGCATCATCGTTGTTC
Itgb3	Forward: GTGAGTGCGATGACTTCTCCTG<br>Reverse: CAGGTGTCAGTGCGTGTAGTAC
Klf4	Forward: CTATGCAGGCTGTGGCAAAACC<br>Reverse: TTGCGGTAGTGCCTGGTCAGTT
Nfkb	Forward: GCTGCCAAAGAAGGACACGACA<br>Reverse: GGCAGGCTATTGCTCATCACAG
Pdgfb	Forward: AATGCTGAGCGACCACTCCATC<br>Reverse: TCGGGTCATGTTCAAGTCCAGC
Pdgfra	Forward: GCAGTTGCCTTACGACTCCAGA<br>Reverse: GGTTTGAGCATCTTCACAGCCAC
Pdgfrb	Forward: GTGGTCCTTACCGTCATCTCTC<br>Reverse: GTGGAGTCGTAAGGCAACTGCA
Pdk1	Forward: CCACTGAGGAAGATCGACAGAC<br>Reverse: AGAGGCGTGATATGGGCAATCC
Tgfb1	Forward: TGATACGCCTGAGTGGCTGTCT<br>Reverse: CACAAGAGCAGTGAGCGCTGA
Tgfbr1	Forward: TGCTCCAAACCACAGAGTAGGC<br>Reverse: CCCAGAACACTAAGCCCATTGC
Tgfbr2	From Qiagen – catalog #QT00135646<br>Sequence is proprietary.

Tnfa	Forward: GGTGCCTATGTCTCAGCCTCTT<br>Reverse: GCCATAGAACTGATGAGAGGGAG

Supplementary Table 1. Primer pair sequences used for quantitative reverse transcription polymerase chain reactions.
