# Supplementary material for: Distinct roles of KLF4 in mesenchymal cell subtypes during lung fibrogenesis
Source: Nat Commun. 2021 Dec 10;12:7179. doi: 10.1038/s41467-021-27499-8 (PMC8664937; doi:10.1038/s41467-021-27499-8)
Supplement: Supplementary file 2 — Description of Additional Supplementary Files [file 41467_2021_27499_MOESM2_ESM.pdf]

Description of Additional Supplementary Files

File Name: Supplementary Data 1
Description: Bulk RNA-sequencing of SMCs treated with siRNA against Klf4 relative to Scr RNA treatment. n=3 independent experiments. DESeq2 was used with two-sided Wald test for p-value and Benjamini-Hochberg FDR for p-adj.

File Name: Supplementary Data 2
Description: Bulk RNA-sequencing of PDGFR-β^+ lung cells treated with siRNA against Klf4 relative to Scr RNA treatment. n=3 independent experiments. DESeq2 was used with two-sided Wald test for p-value and Benjamini-Hochberg FDR for p-adj.

File Name: Supplementary Data 3
Description: Transcription factors predicted by the TRANSFAC database to bind between -5000 bp and +100 bp of Ccl2 gene. Transcription start site of *Ccl2* is at position +1 bp.
