# Supplementary material for: Distinct roles of KLF4 in mesenchymal cell subtypes during lung fibrogenesis
Source: Nat Commun. 2021 Dec 10;12:7179. doi: 10.1038/s41467-021-27499-8 (PMC8664937; doi:10.1038/s41467-021-27499-8)
Supplement: Supplementary file 6 — Reporting Summary [file 41467_2021_27499_MOESM6_ESM.pdf]

nature research

Corresponding author(s): Daniel Greif

Last updated by author(s): Sep 27, 2021

Reporting Summary

Nature Research wishes to improve the reproducibility of the work that we publish. This form provides structure for consistency and transparency in reporting. For further information on Nature Research policies, see our Editorial Policies and the Editorial Policy Checklist.

Statistics

For all statistical analyses, confirm that the following items are present in the figure legend, table legend, main text, or Methods section.

n/a	Confirmed	
☐	☒	The exact sample size (*n*) for each experimental group/condition, given as a discrete number and unit of measurement
☐	☒	A statement on whether measurements were taken from distinct samples or whether the same sample was measured repeatedly
☐	☒	The statistical test(s) used AND whether they are one- or two-sided<br>*Only common tests should be described solely by name; describe more complex techniques in the Methods section.*
☒	☐	A description of all covariates tested
☐	☒	A description of any assumptions or corrections, such as tests of normality and adjustment for multiple comparisons
☐	☒	A full description of the statistical parameters including central tendency (e.g. means) or other basic estimates (e.g. regression coefficient) AND variation (e.g. standard deviation) or associated estimates of uncertainty (e.g. confidence intervals)
☐	☒	For null hypothesis testing, the test statistic (e.g. *F*, *t*, *r*) with confidence intervals, effect sizes, degrees of freedom and *P* value noted<br>*Give P values as exact values whenever suitable.*
☒	☐	For Bayesian analysis, information on the choice of priors and Markov chain Monte Carlo settings
☒	☐	For hierarchical and complex designs, identification of the appropriate level for tests and full reporting of outcomes
☒	☐	Estimates of effect sizes (e.g. Cohen's *d*, Pearson's *r*), indicating how they were calculated

Our web collection on statistics for biologists contains articles on many of the points above.

Software and code

Policy information about availability of computer code

Data collection	scRNA sequencing data was processed with Cell Ranger v3.1.0.
Data analysis	scRNA sequencing data was analyzed using R program version 3.6.1 with the Seurat package v3.1.0, the marker heat map was generated with the R package ComplexHeatmap v3.2.3. For bulk RNA sequencing analysis, signal intensities are converted to individual base calls during a run using the system's Real Time Analysis (RTA) software, the reads were later trimmed to remove low quality base-calls. HISAT2 was used to align the trimmed reads to the reference genome with GENCODE annotation for mouse. Gene counts and transcript abundance were estimated using ballgown/stringTie and differential gene expression analysis was performed using R-based DESeq2 v1.24.0. For image processing, ImageJ v1.52 and Adobe Photoshop v20.0.8 were used. For analysis of flow cytometric data, FlowJo v10.5.0 was used.

For manuscripts utilizing custom algorithms or software that are central to the research but not yet described in published literature, software must be made available to editors and reviewers. We strongly encourage code deposition in a community repository (e.g. GitHub). See the Nature Research guidelines for submitting code & software for further information.

Data

Policy information about availability of data

All manuscripts must include a data availability statement. This statement should provide the following information, where applicable:

- Accession codes, unique identifiers, or web links for publicly available datasets
- A list of figures that have associated raw data
- A description of any restrictions on data availability

The bulk and scRNA-seq data are publicly available in Gene Expression Omnibus (accession code GSE184672: https://www.ncbi.nlm.nih.gov/geo/query/acc.cgi?acc=GSE184672). The TRANSFAC database is publicly available (https://portal.genexplain.com/cgi-bin/portal/login.cgi). Data are available in the main figures and supplementary figures and tables. Trimmed bulk RNA-seq reads were aligned to the reference mouse genome mm10 (https://www.ncbi.nlm.nih.gov/assembly/

GCF_000001635.20/). The source data underlying Figs. 1c, d, f, h, i, 2b, d, e, g, i, 3c-e, g, i, 4a-h, 5c-e, g, i, 6a-f, h, j-l, 7d-l, S5b, S8, S9b, c, S11a, c, S13a, c, S14, S15b are provided as a Source Data file. There is no restrictions on data availability.

Field-specific reporting

Please select the one below that is the best fit for your research. If you are not sure, read the appropriate sections before making your selection.

- [x] Life sciences
- [ ] Behavioural & social sciences
- [ ] Ecological, evolutionary & environmental sciences

For a reference copy of the document with all sections, see nature.com/documents/nr-reporting-summary-flat.pdf

Life sciences study design

All studies must disclose on these points even when the disclosure is negative.

Sample size	The pre-specified statistical significance was set at $p<0.05$. For all studies, the number of mice or repeats of cell culture experiments was not estimated in advance because we could not estimate the variability or effect size. As the studies progressed, we used the number of mice or cell culture experimental repeats that would reach statistical significance. The number of mice and/or experimental repeats are indicated in each figure legend and were generally between 3 to 7 times. The consistent reproducibility of our findings enhanced the statistical power.
Data exclusions	No data were excluded.
Replication	At least n=3 biological repeats were undertaken for experiments. All attempts at replication were successful.
Randomization	Mice were stratified into groups based on their genotype. Within a genotype, mice were randomly selected to receive tamoxifen or vehicle and/or bleomycin or vehicle. For experiments with cultured cells, samples were randomly allocated into experimental groups.
Blinding	Investigators were blinded to group allocation while conducting experiments and analyzing results.

Reporting for specific materials, systems and methods

We require information from authors about some types of materials, experimental systems and methods used in many studies. Here, indicate whether each material, system or method listed is relevant to your study. If you are not sure if a list item applies to your research, read the appropriate section before selecting a response.

Materials & experimental systems

n/a	Involved in the study	
[ ]	[x]	Antibodies
[ ]	[x]	Eukaryotic cell lines
[x]	[ ]	Palaeontology and archaeology
[ ]	[x]	Animals and other organisms
[ ]	[x]	Human research participants
[x]	[ ]	Clinical data
[x]	[ ]	Dual use research of concern

Methods

n/a	Involved in the study	
[x]	[ ]	ChIP-seq
[ ]	[x]	Flow cytometry
[x]	[ ]	MRI-based neuroimaging

Antibodies

Antibodies used	Primary Ab's: Chicken anti-GFP (Abcam, Cat. #ab13970, Lot # GR293362-1); Rabbit anti-KLF4 (Cell Signaling, Cat. # 4038S, Lot #3); Rat anti-CD68 (Biorad, Cat. #MCA1957, Clone #FA-11, Lot #1807); Rat anti-MECA32 (Developmental Studies Hybridoma Bank, Cat. #AB 531797, Isotype: IgG2a); Mouse Cy3-anti-SMA (Sigma-Aldrich, Cat. #C6198, Clone #1A4, Lot #086M4829V); Goat biotinylated anti-PDGFR-beta (R&D, Cat. #BAF1042, Lot #HeJ0317031); Mouse PE-anti-PDGFR-beta (Miltenyi Biotec, Cat. #130-102-501, Clone #APB5, Lot #5171009427); Rabbit anti-SMAD2/3 (Cell Signaling Technology, Cat. #3102, Lot #9), Rabbit anti-GAPDH (Cell Signaling Technology, Cat. #2118S, Lot #14), Rabbit anti-phospho-SMAD3 (Abcam, Cat. #ab52903, Lot #GR128879-79), Rabbit anti-collagen 1 (Abcam, Cat. #ab34710, Lot #GR271609-4), Rabbit anti-CCL2 (Abcam, Cat. #ab25124, Lot #GR1152-57) and Mouse anti-fibronectin (BD Biosciences, Cat. #610077, Clone #10), Rabbit anti-Foxm1 (Abcam, Cat. #ab207298, Lot #GR3279431-1).<br>Secondary Ab's: Goat anti-rabbit HRP (DAKO, Cat. #P0448, Lot. #20079938), Goat anti-mouse HRP (DAKO, Cat. #P0447, Lot. #20078279), Goat anti-chicken Alexa-488 (Abcam, Cat. #ab150169, Lot. #GR2966492), Goat anti-rabbit Alexa-488 (Invitrogen, Cat. #A11008, Lot. #2110498), Goat anti-rabbit Alexa-564 (Invitrogen, Cat. #A32740), Goat anti-rabbit Alexa–647 (Invitrogen, Cat. #A21244, Lot. #1834794), Goat anti-rat Alexa-564 (Invitrogen, Cat. #A21247, Lot. #2268323).
Validation	Chicken anti-GFP – IHC use reported in brain sections (Kerman et al., 2006, J Comp Neurol) and image from olfactory mouse tissue section on Abcam website; Rabbit anti-KLF4 – WB image shown on Cell Signaling Technology website and previously used on human pulmonary artery smooth muscle cells (Sheikh et al., 2015, Sci Transl Med). ChIP in aortic SMCs (Salmon et al., 2019, Physiol Rep). IHC staining of lung gives similar result as with previously published anti-KLF4 Ab from R&D Systems (Sheikh et al., 2015, Sci Transl Med); Rat anti-CD68 - on Bio-Rad website, noted to work in immunohistology and immunofluorescence. For IHC, used in many papers; for instance, on mouse atherosclerotic plaque sections (Riedl et al., 2020, PLoS ONE); Rat anti-MECA32 – extensively used in IHC; for

instance, IHC of mouse brain, heart and skeletal muscle (Halmann et al., 1995, Dev Dynamics) and of lung (Sheikh et al., 2018, Cell Rep); Mouse Cy3-anti SMA – extensively used in IHC; for instance in lung sections (Ntokou et al., 2021, JCI Insight; Greif et al., Dev Cell, 2012); Goat biotinylated anti-Pdgfr-beta - IHC use in the lung (Sheikh et al., 2018, Cell Rep; Greif et al., Dev Cell, 2012); Mouse PE-anti-PDGFR-beta – was validated on Miltenyi Biotec website for flow cytometry; Rabbit anti SMAD2/3 – widely used for WB; for instance on lung epithelial cells (Kyung et al., 2018, BMC Pharmacol Toxicol) and brain sections (Dave et al., 2018, Dev Cell); Rabbit anti-GAPDH – example of WB use on Cell signaling Technology website and widely used in the literature (Dave et al., 2018, Dev Cell; Song et al., 2021, Mol Med Rep); Rabbit anti-phospho-SMAD2/3 – WB use example on mouse epithelial lysates on Abcam website and used in the literature (Dave et al., 2018, Dev Cell); Rabbit anti-collagen 1 – WB use exemplified on Abcam website. It has been used for WB in the literature; for instance on mouse cardiomyocyte lysates (Li et al., 2021, Mol Med Rep); Rabbit anti-CCL2 – WBs shown on customer reviews on the Abcam website illustrate antibody works well. It has been used in the literature for WB (e.g., Li et al., 2021, Aging Cell); Mouse anti-fibronectin – WB use exemplified on epidermal carcinoma cell line on BD Biosciences website. It has been used in the literature for WB (e.g., Chen, 1996, J Biol Chem); Rabbit anti-FoxM1 - WBs exemplified on Abcam website. It has been used in WB in the literature; for instance in lung carcinoma tissue (Li, 2020, Oncology Letters; Lv et al., 2020, Cell Death and Disease).

Eukaryotic cell lines

Policy information about cell lines

Cell line source(s)	Cell Biologics (airway SMCs)
Authentication	Expression of SMC markers was confirmed.
Mycoplasma contamination	Not tested
Commonly misidentified lines (See ICLAC register)	N/A

Animals and other organisms

Policy information about studies involving animals; ARRIVE guidelines recommended for reporting animal research

Laboratory animals	Species: Mus musculus; Strains: ROSA26R(ZsGreen1/ZsGreen1), ROSA26R(mTmG/mTmG) and ROSA26R(YFP/YFP) mice were obtained from Jackson Laboratory. Klf4(flox/flox) mice were from purchased from the Mutant Mouse Resource & Research Center. Acta2-CreERT2, Pdgfrb-CreERT2 and ROSA26R(Rb/Rb) mice were provided by investigators who generated these mice; Sex: male and female; Age and sex-matched experimental and control mice were used. Mice (including ROSA26R(Zs/+) mice] used in experiments were 3-4 months old.
Wild animals	No wild animals were used in the study
Field-collected samples	No field collected samples were used in the study
Ethics oversight	Yale University IACUC

Note that full information on the approval of the study protocol must also be provided in the manuscript.

Human research participants

Policy information about studies involving human research participants

Population characteristics	De-identified lung tissues used in this study had been previously obtained from males and females aged 63-78 years old whose biopsy revealed idiopathic fibrosis (IPF) or from resection of lung nodules that were found to be benign (control).
Recruitment	For this study, The Yale Lung Repository provided formalin fixed, paraffin-embedded slides from banked, de-identified, excess specimens that had been previously obtained with patient consent from clinically indicated biopsies performed on patients followed in the Pulmonary and Thoracic Surgery Programs. Potential sources of bias include the analysis of tissue from patients who required biopsy as the vast majority of patients can be diagnosed with CT scan. Additional sources of bias include this tissue being from a single center and that the controls are not truly "normal" since they were undergoing procedures.
Ethics oversight	Human studies were performed with approval of Human Investigation Committee at Yale University.

Note that full information on the approval of the study protocol must also be provided in the manuscript.

Flow Cytometry

Plots

Confirm that:

- [x] The axis labels state the marker and fluorochrome used (e.g. CD4-FITC).
- [x] The axis scales are clearly visible. Include numbers along axes only for bottom left plot of group (a 'group' is an analysis of identical markers).
- [x] All plots are contour plots with outliers or pseudocolor plots.
- [x] A numerical value for number of cells or percentage (with statistics) is provided.

Methodology

Sample preparation	Lungs were finely minced and incubated in 2 mg/ml collagenase I (Worthington) in PBS at 37C for 20 min. The digestion mixture was passed through a 14-gauge pipetting needle (Cadence Science), incubated at 37C for an additional 20 min and then filtered through a 70 micrometer cell strainer (Falcon). This single cell suspension filtrate was centrifuged at 1200 rpm for 5 min, and the pellet was resuspended in cold PBS with 1% fetal bovine serum (FBS; Invitrogen). The resuspended cells were incubated with either PE-conjugated anti-PDGFR- antibody (1:200, Miltenyi Biotec) for 20 min in the case of Pdgfrb-CreERT2, Klf4(flox/flox) mice or DAPI (1:1000) to label dead cells for 10 min in the case of Pdgfrb-CreERT2, ROSA26R(Zs/+) mice. The sample was then washed in cold PBS with 1% FBS and centrifuged at 1200 rpm for 5 min. The resulting pellet was resuspended in PBS containing 1% FBS and 0.02% EDTA. DAPI (1:2000) was added to cells from Acta2-CreERT2, ROSA26R(Zs/+) mice or cells from Pdgfrb-CreERT2, Klf4(flox/flox) mice that had undergone incubation with anti-PDGFR-beta antibody. Finally, PDGFR-beta+DAPI- or Zs+DAPI- cells were sorted on a BD FACSAria II cell sorter. Cells stained with DAPI alone were used as a control for anti-PDGFR-beta antibody specificity. Lungs from C57BL/6 mice were processed similarly to those of Pdgfrb-CreERT2, ROSA26R(Zs/+) mice, and isolated cells were used as a control for auto-fluorescence in the Zs channel.
Instrument	BD FACSAria II cell sorter
Software	FlowJo v10.5.0
Cell population abundance	PDGFR-beta+ cells comprised ~5-10% of total mouse lung cells. Viable cells were obtained by negative selection for DAPI. For the isolation of PDGFR-beta+ cells from Pdgfrb-CreERT2, Klf4(flox/flox) mice using PE anti-PDGFR-beta antibody, a DAPI control without antibody staining was used to establish gating parameters. In studies with the isolation of Zs+ cells from Pdgfrb-CreERT2, ROSA26R(Zs/+) mice and Acta2-CreERT2, ROSA26R(Zs/+) mice, C57BL/6 mouse lung single cells prepared similarly were used as a control for auto-fluorescence. Purity of the isolated Zs+ cells were determined after sorting as well as after passaging the cells by assessing Zs fluorescence (purity was ~100%).
Gating strategy	1) Gating for the Zs+ cells from Pdgfrb-CreERT2, ROSA26R(Zs/+) and Acta2-CreERT2, ROSA26R(Zs/+) mice were determined by using C57BL/6 lung single cells prepared similarly - shown in Figure S12; 2) Gating for the PDGFR-PE labeled cells were determined with a control lacking antibody staining and the DAPI only control of the same lung preparation. 3) Dead cells in all samples were sorted out by negative selection for DAPI staining.

- [x] Tick this box to confirm that a figure exemplifying the gating strategy is provided in the Supplementary Information.
